# Convergent Approach Toward ADP‐Ribosylated Peptides via a Chemoselective Phosphate Condensation

**DOI:** 10.1002/chem.202501383

**Published:** 2025-06-16

**Authors:** Sven Wijngaarden, Femke L. A. M. van der Heijden, Cindy J. Bogaart, Maria Siskou, Ioanna Tsoumani, Daisy J. Robinson, Gijsbert A. van der Marel, Jeroen D. C. Codée, Herman S. Overkleeft, Dmitri V. Filippov

**Affiliations:** ^1^ Leiden Institute of Chemistry Leiden University Einsteinweg 55 Leiden 2333 CC The Netherlands

**Keywords:** adenosine diphosphate ribose, peptides, phosphate condensation, phosphorylation, solid‐phase synthesis

## Abstract

The preparation of well‐defined ADP‐ribosylated peptides is essential for studying the functional implications of this post‐translational modification. While methodologies exist for the chemical synthesis of short oligo‐ADPr fragments and mono‐ADP‐ribosylated peptides separately, combining the two distinct chemistries, required to assemble them, has remained challenging. In this research, we employ a methodology for the regio‐ and chemoselective condensation of two phosphomonoesters to convergently install the pyrophosphate bond in ADP‐ribosylated constructs. A diverse set of phosphoribosylated peptides, varying in the amino acid acceptor, was prepared and condensed with adenosine monophosphate to yield mono‐ADP‐ribosylated peptides. Furthermore, a solid‐phase approach was developed to access a phosphoadenosyl‐ADPr fragment, which was used to prepare a di‐ADPr‐peptide as the first example of a synthetic oligo‐ADP‐ribosylated peptide. Overall, the work presented here extends synthetic methodology for the preparation of well‐defined ADP‐ribosylated peptides.

## Graphical abstract

This research presents a convergent approach for synthesizing well‐defined ADP‐ribosylated peptides. It involves a regio‐ and chemoselective condensation of phosphoadenosyl (ADPr) fragments with phosphoribosyl peptides, allowing for the creation of various ADP‐ribosylated peptides, including a di‐ADP‐ribosylated‐peptide as the first example of a synthetic oligo‐ADP‐ribosylated peptide.

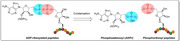

## 1 Introduction

Adenosine diphosphate ribosylation (ADP‐ribosylation) is a widespread post‐translational modification of which the functional role in molecular biology remains poorly understood.^[^ ^1^ ^]^ Transferase enzymes (ARTs, PARPs) glycosylate various amino acid side chains, using NAD^+^ as the glycosyl donor, generating mono‐ADP‐ribosylated proteins. Subsequent iterative PARP‐mediated glycosylation of the 2′‐OH of adenosine in ADP‐ribosyl proteins leads to the formation of sugar‐pyrophosphate chains known as poly‐ADPr or PAR. Many amino acid side chains can be modified with ADP‐ribose, and the reported acceptor sites include serine, glutamic acid, aspartic acid, arginine, tyrosine, cysteine, and histidine.^[^ ^2^, ^3^ ^]^ Although the majority of these sites are thought to be modified with mono‐ADPr exclusively, poly‐ADP‐ribosylation has been established for serine^[^ ^4^, ^5^ ^]^ and glutamic acid^[^ ^6^, ^7^ ^]^ side chains. The ADP‐ribosylation of proteins is regulated by specific hydrolases such as PARG that degrades the ADP‐ribose chain and ARH1 and ARH3 that cleave the glycosidic bond between the “distal” ribose in ADPr and the amino acid side chains.^[^ ^8^, ^9^, ^10^, ^11^, ^12^ ^]^ Access to synthetic peptides modified with ADP‐ribose chains of defined lengths would be invaluable to better understand the biosynthetic routes and signaling pathways involving ADP‐ribose as post‐translational modification. ADP‐ribosylated peptides and proteins, such as core histones and other chromatin architecture proteins, prepared by chemo‐enzymatic methods and containing short stretches of oligo‐ADP‐ribose, have already provided insights into the impact of the ADPr‐chain length attached to a serine side chain on the chromatin structure at DNA damage sites.^[^ ^13^, ^14^, ^15^, ^16^ ^]^

Over the past two decades, significant advances have been made in the chemical synthesis of well‐defined ADP‐ribosylated peptides. Our approach for the synthesis of mono‐ADPr‐peptide comprises the incorporation of preribosylated amino acid building blocks in a solid‐phase peptide synthesis (SPPS), followed by on‐resin phosphorylation and pyrophosphorylation via P(V)–P(III) phosphate‐phosphoramidite chemistry.^[^ ^17^ ^]^ This method enabled the production of ADP‐ribosylated peptides of almost all biologically relevant amino acid acceptor sites.^[^ ^18^, ^19^, ^20^ ^]^ However, the challenges in combining the protection schemes for the peptide and ADPr‐moiety remain, for example, in the case of the very acid‐sensitive phenolic glycosyl bond of Tyr‐ADPr.^[^ ^21^ ^]^ As well, we prepared, “free” oligo‐ADP‐ribose fragments up to the pentamer on a solid‐support using P(III)–P(V) chemistry for the installation of the inter‐ribose pyrophosphate functionality.^[^ ^22^, ^23^, ^24^ ^]^ Alternatively, Lambrecht et al. employed a solution‐phase approach for the preparation of an ADPr‐dimer via a synthetic route that relied on a P(V)‐based phosphorimidazolide‐phosphate coupling.^[^ ^25^ ^]^

**Scheme 1:**
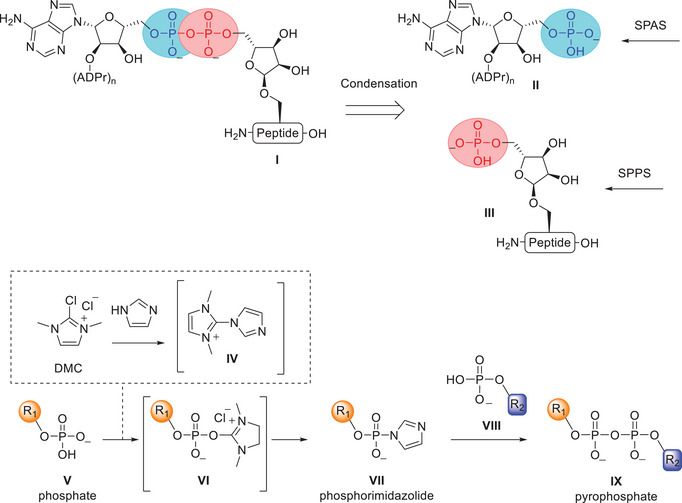
*Above*: Retrosynthetic analysis for the convergent synthesis of ADP‐ribosylated peptides (**I**). Disconnection of ADPr‐peptide **I** at the peptide proximal pyrophosphate linkage, produces two phosphates which are chemo‐ and regioselectively condensed. Phosphates **II** and **III** are accessed via solid‐phase ADPr synthesis (SPAS) and solid‐phase peptide synthesis (SPPS). *Below*: General reaction scheme of the phosphorimidazolide‐based methodology for producing pyrophosphates. Phosphate activation via dehydrating reagent **IV** and subsequent pyrophosphorylation. Imidazole and DMC give dehydrating reagent **IV** that dehydrates phosphate **V** to produce electrophile **VI**. Nucleophilic catalysis by imidazole gives phosphorimidazolide **VII**, which can pyrophosphorylate phosphate **VIII** to produce pyrophosphate **IX**.^[^ ^26^ ^]^

Existing synthetic methods for ADPr‐peptides and poly‐ADPr fragments rely on two distinct solid‐phase approaches, which are intrinsically hard to combine. The difficulties arise from the necessity to develop protective group strategies for all the amino acid side chains, which would be incompatible with the presence of acid‐sensitive oligo‐ADP‐ribose in the final construct. In this work, we apply a convergent strategy that avoids this potential incompatibility by chemoselective ligation of phosphomonoesters to install the pyrophosphate at the final stage of the synthesis of ADPr‐peptides (Scheme 1). This approach involves retrosynthetic disconnection of ADP‐ribosyl peptide **I** at the peptide proximal pyrophosphate bond, generating two key phosphate components: an ADP‐ribose fragment **II** lacking the “distal” 5′'‐O‐phosphoribosyl moiety and a phosphoribosyl‐functionalized peptide (Pr‐peptide) **III**. Fully deprotected and purified ADPr fragment **II** is accessed via solid‐phase ADPr synthesis (SPAS), while the phosphoribosyl‐peptide **III** can be obtained through standard SPPS. Fragments **II** and **III** are then coupled through a suitable phosphate condensation. We opted here for methodology that relies on the coupling of phosphate nucleophiles with phosphorimidazolide electrophiles, which offers notable chemo‐ and regioselectivity^[^ ^27^, ^28^ ^]^ and has been shown to be compatible with aqueous conditions^[^ ^26^ ^]^ required to dissolve unprotected peptides and ADP‐ribose fragments (Scheme 1). The method of choice generates phosphorimidazolides via selective dehydration of phosphates using 2‐chloro‐1,3‐dimethylimidazolinium chloride (DMC) and imidazole (Scheme 1). This reaction proceeds via formation of imidazolinium species **IV**, which can selectively dehydrate phosphates such as **V** via intermediate **VI** to generate phosphorimidazolide **VII**.^[^ ^26^, ^29^ ^]^ Nucleophilic attack by phosphate **VIII** on phosphorimidazolide **VII** then yields pyrophosphate diester **IX**. There is ample literature precedent for the application of this phosphorimidazolide‐based methodology in both organic solvents and aqueous media for the synthesis of NDP‐sugars,^[^ ^26^, ^30^ ^]^ 5′,5′‐oligophosphorylated dinucleosides,^[^ ^29^, ^31^, ^32^, ^33^ ^]^ pyrophosphorylated peptides,^[^ ^34^ ^]^ proteins,^[^ ^35^ ^]^ and simple ADPr‐peptides.^[^ ^36^ ^]^ Catalysis with metal salts, such as zinc‐ or magnesium chloride has proven essential to enhance reactivity and selectivity of the phosphate–phosphate condensation.^[^ ^26^, ^37^ ^]^

In this work, we first evaluated the compatibility of the phosphate condensation reaction with a range of ADP‐peptides modified on serine, cysteine, tyrosine, and arginine. Hereafter, a phosphoadenosyl‐ADPr fragment, containing the 5′‐O‐phosphate, was prepared and condensed to a phosphoribosyl peptide, to produce for the first time a di‐ADPr‐peptide via a fully synthetic approach.

## 2 Results and Discussion

Phosphoribosyl‐peptides bearing an O‐, S‐, or N‐glycosidic linkage (serine, cysteine, tyrosine, and arginine) were accessed by SPPS (Scheme S1 and S2). These peptides were prepared by adaptation of established protocols, via incorporation of their corresponding ribofuranosylated building blocks, followed by on‐resin phosphorylation.^[^ ^21^, ^38^, ^39^, ^40^, ^41^ ^]^ The peptide sequences were derived from proteins known to be ADP‐ribosylated on the indicated amino acids (Table S1).

Hereafter, the coupling of AMP‐imidazolide (AMP‐Im) to seryl‐phosphoribose peptide **2** to produce mono‐ADPr‐peptide **6** was examined (Table 1). Key parameters assessed included stoichiometry, concentration, and the effect of additives on the phosphate condensation reaction. AMP was reacted with DMC and imidazole in a molar ratio of 1:2:4 to form the activated AMP‐Im in 30 minutes (Figure S1). Condensation using 5 eq. of AMP‐Im, 20 eq. of ZnCl_2_ as a Lewis acid, and a final phosphoribosyl‐peptide concentration of 8 mM, gave 37% conversion after 5 hours at 40 °C (Table 1, entry 1), based on UV integration after LCMS analysis. The most prominent side product, as identified by MS, was the symmetrical pyrophosphate **AppA**. Increasing the amount of zinc chloride from 20 to 100 equivalents improved conversion to 52% (entry 2).^[^ ^37^ ^]^ Increasing the concentration of phosphoribosyl‐peptide **2** from 8 mM to 25 mM further improved conversion to 67% (entry 3). Notably, no evidence of homodimerization of phosphoribosyl‐peptide **2** was found in this last experiment. Combining both optimizations (higher peptide concentration and increased amount of zinc chloride) further improved conversion to 79% (entry 4). The reaction progress was followed over time by LCMS analysis for the conditions stated in entry 4, which showed full consumption of **AMP‐Im** after 5 hours (Figure S3). The reaction progress likely halts due to competing hydrolysis of the phosphorimidazolide. Therefore, to further improve the reaction conversion, an additional 5 equivalents of phosphorimidazolide AMP‐Im were added after 2.5 hours, leading to 77% conversion (entry 5). Finally, increasing the concentration of zinc chloride to 100 equivalents resulted in near quantitative conversion of 95% to mono‐ADPr‐peptide **6** (entry 6).

**Table 1:** Optimization of reaction conditions for the phosphate condensation reaction to produce mono‐ADPr‐peptide **6**.

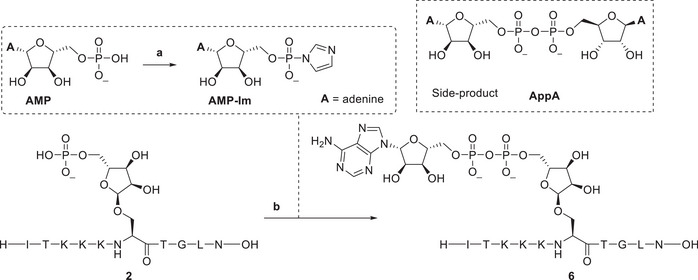				
Entry	Eq. AMP‐Im	Concentration [2] [mM]	Eq. ZnCl_2_	Conversion^[a]^
1	5	8	20	37%
2	5	8	100	52%
3	5	25	20	67%
4	5	25	100	79%
5	2 × 5	25	20	77%
6	2 × 5	25	100	95%

Reagents and conditions: **a**) 20 eq. imidazole, 10 eq. DMC, D_2_O, 40 °C, 45 minutes. **b**) AMP‐imidazolide, ZnCl_2_, 40 °C, 5 hours.

^[a]^ Activation of AMP to AMP‐Im was performed at 200 mM in D_2_O at 40 °C for 45 minutes. Test reactions were performed on a scale of 1 µmol at 40 °C and analyzed after 5 hours by LCMS. Conversion was based on UV integration at 200 nm of the peaks corresponding to the Pr‐peptide **2** and the ADPr‐peptide **6** (Figure S2).

Using the conditions described in Table 1 (entry 6), a diverse set of phosphoribosylated peptides was condensed with commercially available AMP to produce the corresponding ADP‐ribosylated peptides (**6**–**9**, Table 2, Table S1). Reactions were conducted on a scale of 1–5 µmol, after which the ADPr‐peptides were isolated via size exclusion chromatography, followed by reversed‐phase HPLC. The reaction of serine phosphoribosyl‐peptide **2** produced ADPr‐peptide **6** with a conversion of 90%, consistent with the previously described optimization (Table 2, entry 1). Cysteinyl‐ADPr‐peptide **7** was obtained by condensing phosphoribosyl‐peptide **3** with AMP, achieving a 59% conversion, and a 20% isolated yield (entry 2). Despite the affinity of thioglycosides for Lewis acids such as zinc chloride,^[^ ^42^ ^]^ no significant degradation or adverse effects on the coupling reaction were observed. Pyrophosphorylation of tyrosyl‐phosphoribosyl peptide **4** with AMP to produce ADPr‐peptide **8** proceeded in 75% and peptide **8** was isolated in a yield of 13% (entry 3). Lastly, arginyl‐phosphoribosyl‐peptide **5** was transformed into ADPr‐peptide **9** with a conversion of 63% based on LCMS analysis and was isolated in a yield of 28%. It is important to note that the difference observed between reaction conversions and isolated yields for peptides **6**–**9** can be attributed to the two‐step purification procedure combined with a small reaction scale of 1–5 µmol. Overall, the successful synthesis of mono‐ADP‐ribosyl‐peptides **6**–**9** demonstrates the broad applicability of phosphorimidazolide‐assisted phosphate condensation reaction for the synthesis of ADP‐ribose‐containing biopolymers, with nearly all relevant ADPr‐amino acid acceptors being tolerated in the reaction.

**Table 2:** Chemoselective condensation of phosphoribosyl peptides **2**–**5** to AMP to produce mono‐ADPr‐peptides **6**–**9**.

Entry	ADPr‐peptide	Linkage Type	Scale [µmol]	Conversion^[a]^	Isolated Yield
1	**6**	Ser	5	90%	10%
2	**7**	Cys	1	59%	20%
3	**8**	Tyr	5	75%	13%
5	**9**	Arg	1	63%	28%

Activation of AMP to AMP‐Im was performed at a final concentration of 200 mM in D_2_O at 40 °C for 45 minutes. Coupling reactions were performed with 2 × 5 eq. of AMP‐Im, 100 eq. ZnCl_2_ at 40 °C for 5 hours.

^[a]^ Conversion of the phosphoribose‐peptide to the ADPr‐peptide was based on UV integration at 200 nm obtained from the LCMS spectrum after 5 hours of reaction time (Figure S4).

**Scheme 2A:**
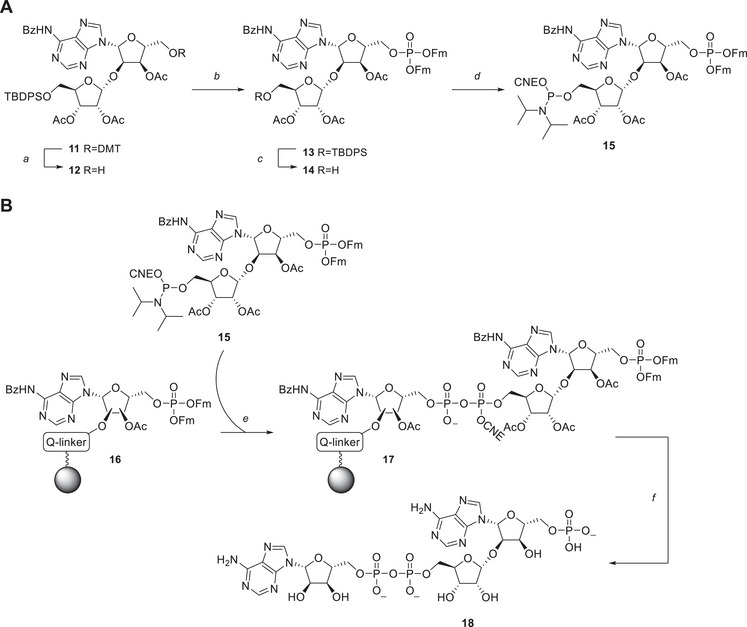
Synthesis of advanced phosphoramidite **15**. Reagents and conditions: A) TFA, DCM, rt, 10 minutes, 63%. B) MS 3 Å, DCI, (FmO)_2_PN(iPr)_2_ **20**,^[^ ^43^ ^]^ ACN, rt, 10 minutes; *then* tBuOOH, decane, 0 °C, 30 minutes, 92% over 2 steps. C) HF·pyridine, THF, 0 °C → rt, 2 hours, 88%. D) DiPEA, 2‐cyanoethyl *N,N*‐diisopropylchlorophosphoramidite, DMF, rt, 10 minutes, 69%. **B**. Synthesis of phosphoadenosyl ADP‐ribose fragment **18**. E) *i*. DBU, ACN, rt, 5 minutes (4x). *ii*. **15**, ETT, ACN, rt, 10 minutes (3x). *iii*. CSO, ACN, rt, 5 minutes (2x). F) *i*. DBU, ACN, rt, 5 minutes (4x). *ii*. NH_4_OH, rt, overnight, 14%.

**Scheme 3:**
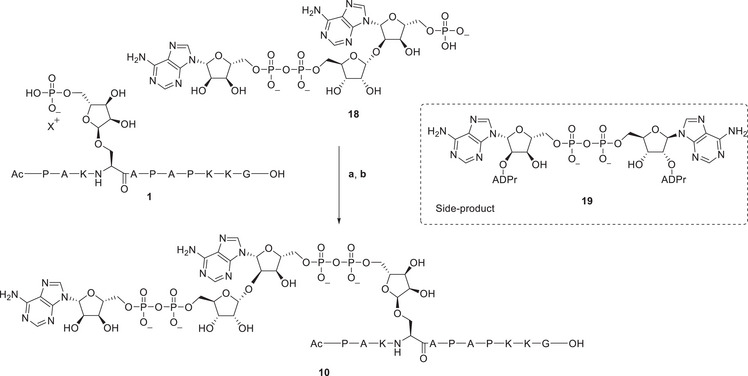
Convergent synthesis of di‐ADPr‐peptide **10** via the chemoselective phosphate condensation reaction. Phosphoribosyl‐ADPr **18** was activated to its corresponding phosphorimidazolide and coupled with phosphoribosyl peptide **1** to produce di‐ADPr‐peptide **10**. Reagents and conditions: **a**) phosphoribosyl‐ADPr **18**, 20 eq. imidazole, 10 eq. DMC, D_2_O, 40 °C, 45 minutes. **b**) 1.2 eq. peptide **1**. 20 eq. ZnCl_2_, D_2_O, 40 °C, overnight, **10**: 12%.

For the condensation reaction toward oligo‐ADP‐ribosylated peptides, first a 1′'‐adenyl‐2′‐yl‐ADPr fragment (phoshoadenosyl‐ADPr **18**) had to be prepared, via a novel solid‐phase approach using P(III)–P(V) phosphoramidite chemistry. The strategy resembles our previously developed method of oligo‐ADPr synthesis^[^ ^23^, ^24^ ^]^ but differs in the direction of assembly, allowing for the straightforward solid‐phase preparation of ADPr with a terminal 5′‐phosphoryladenosine. To this end, advanced adenosyl‐ribose building block **15**, bearing a phosphotriester and phosphoramidite was designed and synthesized starting from orthogonally protected **11** (Schemes 2A, 3 for synthesis of **11**).^[^ ^24^ ^]^ The dimethoxytrityl (DMT)‐ether in **11** was removed using TFA, yielding the primary hydroxyl in **12** in a 63% yield, which was subsequently phosphitylated using difluorenylmethyl N,N‐diisopropylphosphoramidite **20** ^[^ ^43^ ^]^ and 4,5‐dicyanoimidazole (DCI) as the activator. The resulting P(III) phosphite was oxidized using *t*BuOOH, yielding the 9‐fluorenylmethyl (Fm) protected phosphotriester **13** in 92% over two steps. Subsequently, the TBDPS‐ether was removed using acidic HF·pyridine, resulting in alcohol **14** in 88% yield, after which the 5′’‐hydroxyl group was phosphitylated using 2‐cyanoethyl (CNE) N,N‐diisopropylchlorophosphoramidite, leading to the formation of key parobiosyladenine phosphoramidite **15** with a 69% yield.

With the availability of advanced phosphoramidite **15**, a solid‐phase synthesis of phosphoadenosyl‐ADPr fragment **18** was attempted (Scheme 2B). To this end, immobilized and protected adenosine monophosphate **16** was prepared, which was linked to the solid support via the alkali‐sensitive hydroquinone‐*O*,*O*'‐diacetic acid (Q‐linker) (Scheme S4).^[^ ^44^ ^]^ The Q‐linker was selected over conventional linkers due to its enhanced stability toward the base used in this solid‐phase synthesis strategy. The solid‐phase synthesis involved a three‐step procedure, which commenced with the 1,8‐diazabicyclo[5.4.0]undec‐7‐ene (DBU) mediated elimination of the Fm‐protections in phosphotriester **16** to produce the corresponding phosphomonoester. The phosphate was coupled to ribosyladenosine phosphoramidite **17** under activation of 5‐(ethylthio)‐1H‐tetrazole (ETT), producing the cyanoethyl protected P(III)–P(V) species, which was oxidized using camphorsulfonyl‐oxaziridine (CSO) to the P(V)‐P(V) pyrophosphotriester **17**. Finally, removal of the Fm‐ and CNE esters by treatment with DBU, followed by aminolysis of the residuary acetyl and benzoyl protective groups and cleavage of the solid support, produced the AMP‐ADP‐ribose **18** in 14% yield.

**Figure 1:**
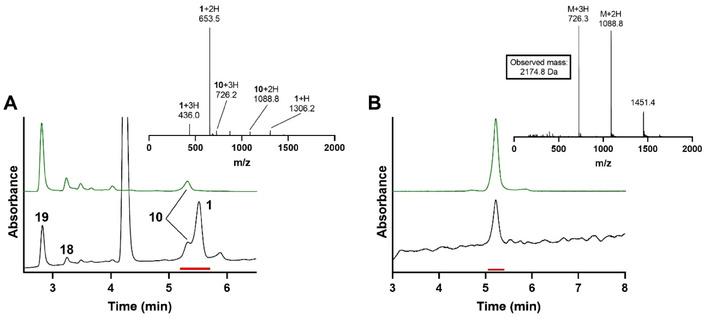
A) Analysis of the condensation reaction toward di‐ADPr‐peptide **10**. Analytical HPLC (linear gradient 00–20% ACN over 10 minutes) of di‐ADPr‐peptide **10**. ESI mass spectrum of the peaks under the red line (5.2–5.7 minutes). Ionization patterns of **1** and **10** are depicted. B) Analytical HPLC (linear gradient 00–20% ACN over 10 minutes) of purified peptide **10**. ESI mass spectrum of the peaks under the red line (5.1–5.5 minutes). Expected mass for di‐ADPr‐peptide **10** is 2174.8 Da. Graphs in black depict the total absorbance measured from 220–680 nm, graphs in green the absorbance of adenine (260 nm).

With the preparation of phosphoadenosyl‐ADPr fragment **18** completed, its chemoselective phosphate condensation to seryl‐phosphoribose peptide **1**, yielding di‐ADPr‐peptide **10**, was investigated (Scheme 3). Given the complex synthesis of poly‐ADPr fragment **18**, initial efforts focused on activating the more synthetically accessible phosphoribosyl peptide **1** as its phosphorimidazolide. However, activation of the phosphoribosyl‐peptide yielded a suspension with no detectable product formation. Therefore, it was decided to activate phosphoribosyl‐ADPr fragment **18** instead. The resulting phosphorimidazolide was then reacted in a small stoichiometric excess with the phosphate of peptide **1**. The amount of zinc chloride used in the synthesis of mono‐ADPr‐peptides was reduced from 100 to 20 equivalents to maintain stoichiometric consistency. The reaction proceeded overnight to ensure complete consumption of the phosphorimidazolide, yielding di‐ADPr‐peptide **10** in a reasonable conversion, as determined by UV integration after LCMS analysis (Figure 1). The most abundant side product which could be identified, originated from homodimerization of phosphoribosyl‐ADPr **18**, forming symmetrical pyrophosphate **19**. After the reaction, the crude mixture was desalted by size exclusion chromatography and purified by ion exchange chromatography affording di‐ADPr‐peptide **10** in 12% yield. The successful isolation of di‐ADPr‐peptide **10** suggests a compatibility of the phosphate coupling reaction with larger, more complex ADPr‐substrates containing multiple pyrophosphate moieties.

## 3 Conclusion

This work describes a convergent synthesis strategy for preparing ADP‐ribosylated peptides via a chemoselective phosphate condensation reaction. The regio‐ and chemoselectivity of the reaction eliminates the need for protecting functional groups, allowing for the late‐stage formation of the peptide proximal pyrophosphate bond. The condensation reaction demonstrates broad applicability for preparing mono‐ADP‐ribosylated peptides modified on various amino acid acceptors. Additionally, a solid‐phase synthetic approach was developed to access phosphoadenosyl‐ADPr fragments, enabling the first chemical synthesis of a di‐ADPr‐peptide, which serves as a model for oligo‐ADP‐ribosylated substrates. Overall, this strategy provides a valuable addition to the chemical toolbox for studying ADP‐ribosylation.

## Supporting Information

The detailed experimental procedures, spectroscopic, and chromatographic characterization.

The authors have cited an additional reference within the Supporting Information.^[^ ^45^ ^]^

## Conflict of Interest

The authors declare no conflict of interest.

## Supporting information



Supporting Information

## Data Availability

The data that support the findings of this study are available in the supplementary material of this article.
