# Supplementary material for: Convergent Approach Toward ADP‐Ribosylated Peptides via a Chemoselective Phosphate Condensation
Source: Chemistry. 2025 Jun 16;31(38):e202501383. doi: 10.1002/chem.202501383 (PMC12238907; doi:10.1002/chem.202501383)
Supplement: Supplementary file 1 — Supporting Information [file CHEM-31-e202501383-s001.docx]

**Table of Contents**

1. Supplementary Tables, Schemes, and Figures S2
2. Experimental Section S8
3. Characterization Data S23

**1. Supplementary Tables, Schemes, and Figures**

**Table S1** Overview of Pr- and ADPr-peptides.

| Code | Parent protein | Modification site | Sequence |
| --- | --- | --- | --- |
| Pr-peptides | | | |
| 1 | H2B1C | Ser 6 | Ac-PAK**S**^Pr^APAPKG-OH |
| 2 | HPF1 | Ser 97 | H-TKKK**S**^Pr^TGLN-OH |
| 3 | PARP7 | Cys 721 | Ac-QEDF**C**^Pr^FLSS-OH |
| 4 | CBX4 | Tyr 207 | Ac-AAKG**Y**^Pr^LGAV-OH |
| 5 | rS1 | Arg 139 | Ac-V**R**^Pr^RV-NH_2_ |
| Mono-ADPr-peptides | | | |
| 6 | HPF1 | Ser 97 | H-TKKK**S**^ADPr^TGLN-OH |
| 7 | PARP7 | Cys 721 | Ac-QEDF**C**^ADPr^FLSS-OH |
| 8 | CBX4 | Tyr 207 | Ac-AAKG**Y**^ADPr^LGAV-OH |
| 9 | rS1 | Arg 139 | Ac-V**R**^ADPr^RV-NH_2_ |
| Poly-ADPr-peptide | | | |
| 10 | H2B1C | Ser 6 | Ac-PAK**S**^di-ADPr^APAPKG-OH |

Synthesis of phosphoribosylated peptides

Phosphoribosylated peptides bearing an *O*-, *S*- or *N*-glycosidic linkage (serine, cysteine, tyrosine and arginine) were accessed by incorporating their corresponding ribofuranosylated building blocks into the desired peptides sequence by SPPS (**Scheme 1** and **Scheme 2**). ^[21,38-40]^

**Scheme S1** Synthetic scheme for the preparation of serine, cysteine and tyrosine phosphoribosyl-peptides **1**-**4**. Pr-peptides were prepared using SPPS starting from Fmoc-building blocks **22** for Pr-Ser, ^[38,39]^ **23** for Pr-Cys ^[39]^ and **24** for Pr-Tyr. ^[21]^ Protecting groups depicted in red were cleaved by acid, in blue by base and in purple by a fluoride source.

**Scheme S2** Synthetic scheme for the preparation of arginyl phosphoribose peptide **5**. ^[16]^ Protecting groups depicted in red were cleaved by acid, in blue by base, in purple by a fluoride source and in orange by palladium mediated deallylation.

Synthesis of building blocks for the preparation of phosphoadenosyl-ADPr

**Scheme S3** Synthesis of orthogonally protected building block **11** for the synthesis of phosphoadenosyl-ADPr. Reagents and conditions: a) **39** was synthesized in 2 steps according to the procedures of Kistemaker *et al.* ^[45]^b) PMB-Br, NaH, DMF/THF, 0 °C → rt, overnight, 89%. c) [Ir(COD)(PMePh_2_)_2_]PF_6_, H_2_, THF, rt; *then* **40**, THF, rt, 2 days; *then* sat. aq. NaHCO_3_, I_2_, rt, 30 min, 86%. d) PTFAI-Cl, Cs_2_CO_3_, acetone, rt, 3 h, 92%. e) 1,3,5-tri-*O*-benzoyl-α-D-ribofuranose **43**, MS 3Å, DCM, rt, 1 h; *then* TMSOTf, -78 °C, 10 min, 77%. f) TFA, DCM, rt, 1 h, 79%. g) DMAP, Ac_2_O, pyridine, rt, 3.5 h, quant. h) *N*^6^-benzoyladenine **47**, BSTFA, ACN, rt, 30 min; *then* HClO_4_-SiO_2_, reflux, overnight, 66%. i) NaOH, pyridine/EtOH/H_2_O, 0 °C, 1 h, 92%. j) DMTCl, pyridine, rt, 2 h; *then* Ac_2_O, 0 °C, 5 h, 90% over 2 steps.

**Scheme S4** Synthesis of immobilized and protected adenosine monophosphate **16** for the synthesis of phosphoadenosyl-ADPr. Reagents and conditions: a) Ac_2_O, pyridine, 0 °C → rt, overnight, 42% for **51A** and **51B**, 22% for **52**. b) DMAP, EDC·HCl, Et_3_N, Q-linker, pyridine, rt, overnight, 46%. c) PyAOP, DiPEA, DMF, rt, 10 min; *then* aminopropyl-CPG, DMF, rt, overnight, 54%. d) *i.* TCA, DCM, rt. *ii.* ETT, (FmO)_2_PN(*i*Pr)_2_ **21**  ^[43]^ , ACN, rt, 10 min (3x). *iii.* CSO, ACN, rt, 5 min (2x).

Supplementary figures


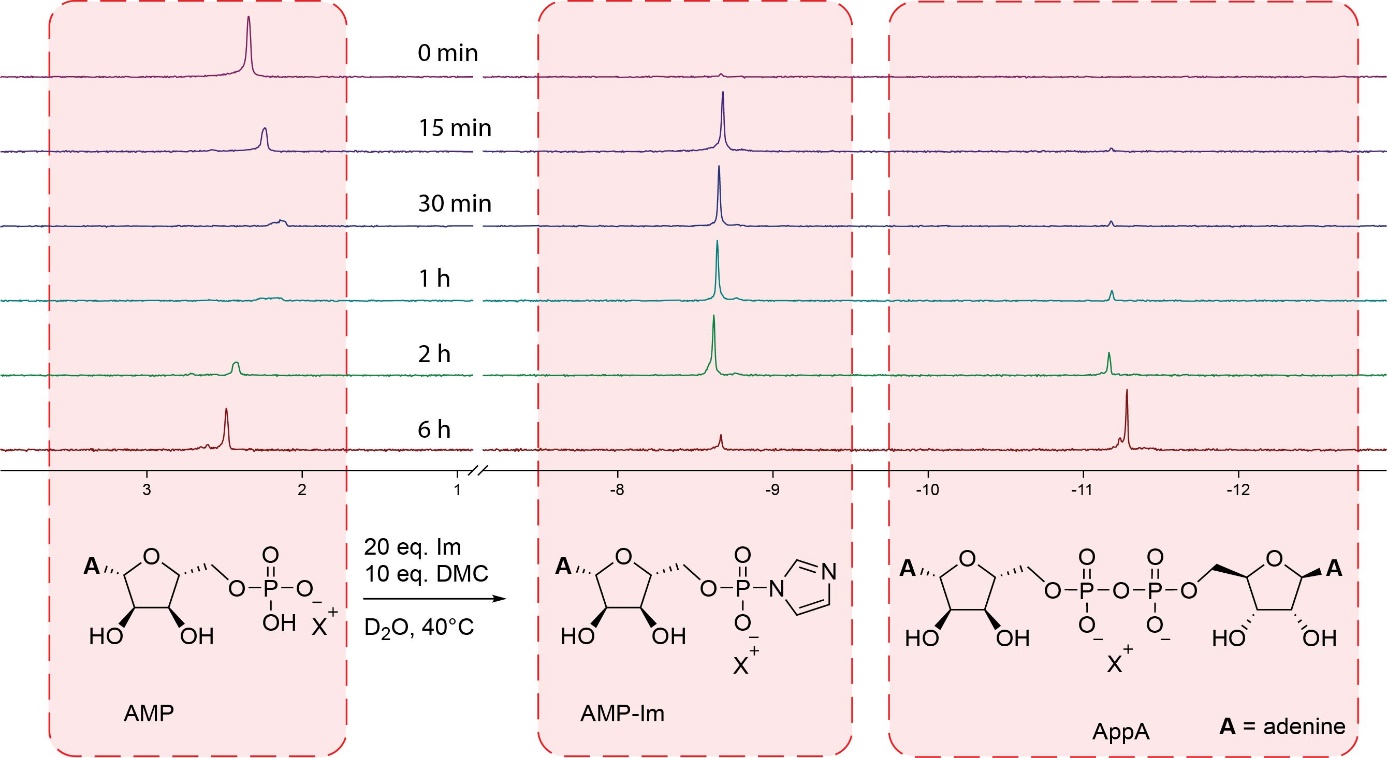


**Figure S1** Activation of AMP to AMP-Im followed monitored over time by ^31^P-NMR. The dehydration reaction was performed at 40 °C. Samples were taken from the activation cocktail and diluted with D_2_O at each indicated time point. The peak at 1 ppm corresponds to AMP, at -6 ppm to AMP-Im and at -11 ppm to symmetrical pyrophosphate AppA.


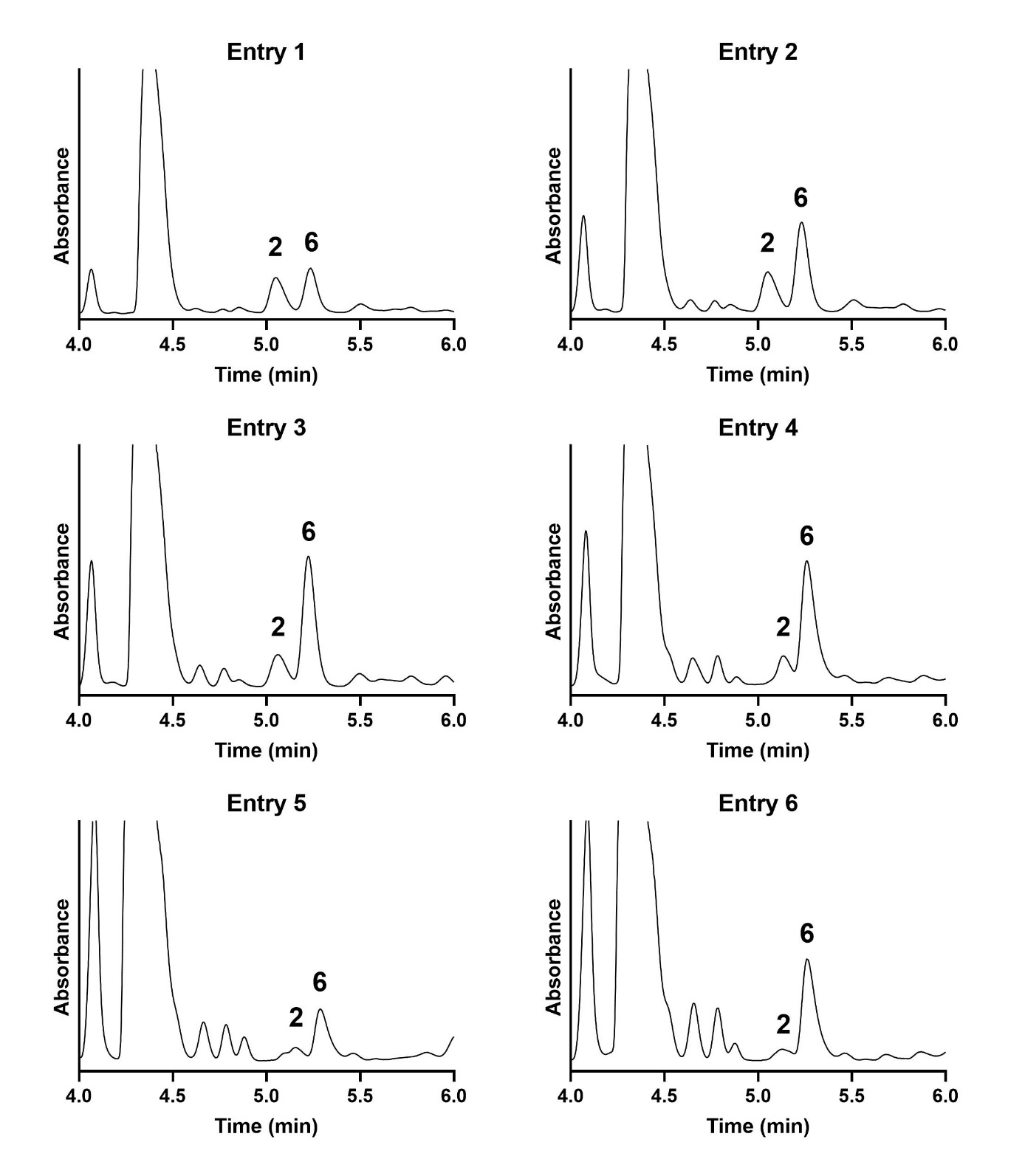


**Figure S2** Analytical LCMS (linear gradient 0-20% ACN over 10 minutes) of crude reaction mixtures obtained from optimization of the phosphate condensation reaction in **Table 1**. Total scan chromatogram of UV absorption from 200-600 nm.


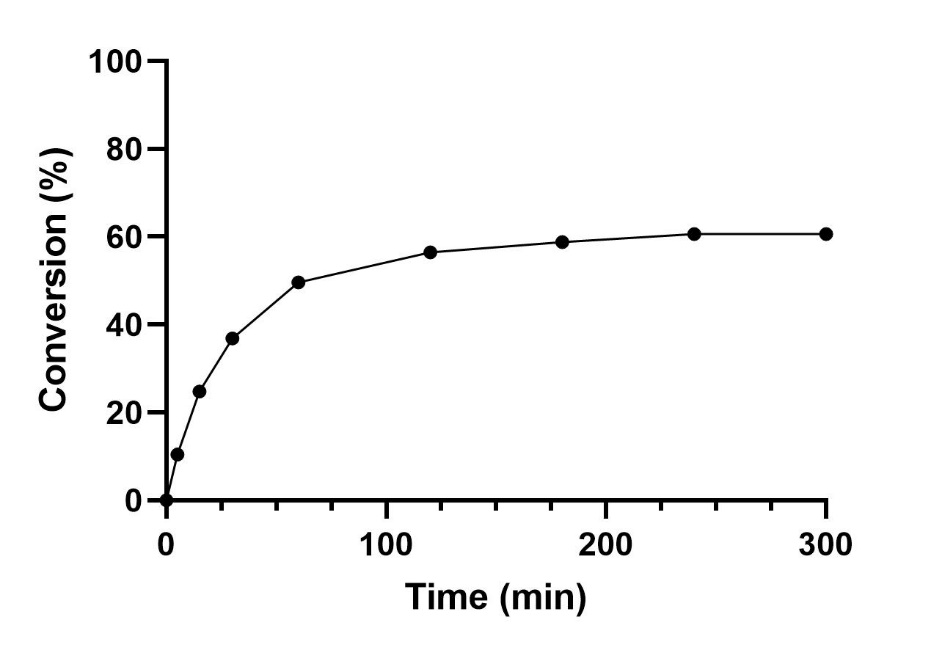


**Figure S3.** Reaction progress of Entry 4, **Table 1** followed over time. Conversion was based on UV integration at 214 nm of the peaks corresponding to the Pr-peptide **2** and the ADPr-peptide **6**.


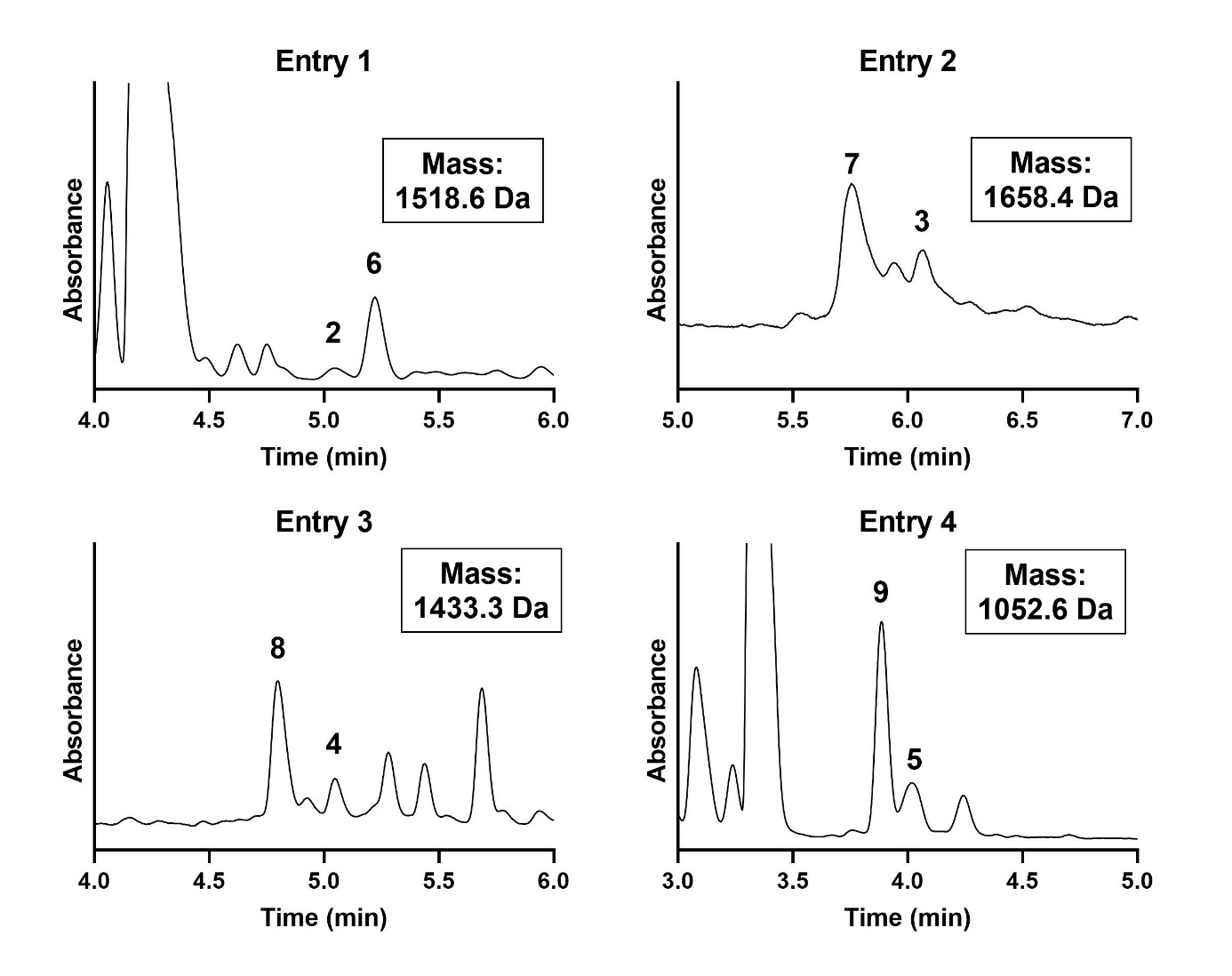


**Figure S4** Analytical LCMS of crude reaction mixtures obtained from reactions towards ADP-ribosylated peptides from **Table 2**. The observed mass corresponding to the ADPr-peptide is indicated.

**2. Experimental Section**

General synthetic procedures

All reagents were of commercial grade and used as received unless stated otherwise. ^1^H- and ^13^C-NMR spectra were recorded on a Bruker AV-400, AV-500 or AV-600 NMR. Chemical shifts (δ) are given in ppm relative to tetramethylsilane as internal standard. Coupling constants (J) are given in Hz. For pyrophosphate-containing compounds, a small amount of EDTA was added to the NMR sample to sharpen the peaks for ^31^P-NMR. All given ^13^C-APT spectra are proton decoupled and are presented with even signals (Cq. and CH_2_) pointing upwards and odd signals (CH and CH3) pointing downwards. LC-MS analysis was performed on a Finnigan Surveyor HPLC system with a Nucleodur C18 Gravity 3 μm 50 x 4.60 mm column (detection at 200-600 nm) coupled to a Finnigan LCQ Advantage Max mass spectrometer with ESI or a Thermo Scientific Vanquish UHPLC coupled to a Thermo Scientific LCQ Fleet ion mass spectrometer with ESI. Buffers used were A= H_2_O, B= MeCN and C= 1% TFA/H_2_O. The methods used were 10→90% 13.5 min (0→0.5 min: 10% MeCN; 0.5→8.5 min: 10% to 90% MeCN; 8.5→11 min: 90% MeCN; 11→13.5 min: 10% MeCN) or 0→50% 13.5 min. HPLC purification was performed on a Gilson GX-281 preparative HPLC with a Gemini-NX 5u, C18, 110 Å, 250 x 10.0 mm column or on a Waters autopurifier HPLC/MS system coupled to a Phenomenex Gemini 5μm 150x21.2 mm column. Buffers used were A= 1% AcOH/H_2_O, B= 10% H_2_O/MeCN. HRMS was recorded on a Thermo Scientific Q Exactive HF Orbitrap mass spectrometer equipped with an electrospray ion source. For HW-40 gel filtration purification, an ÄKTA explorer system equipped with a Superdex-30-HR column (16 mm x 100 cm, flow 1 mL/min) was used.

General procedure A: peptide synthesis

The amino acids (obtained from Novabiochem and Sigma Aldrich) applied in the synthesis were: Fmoc-Ala-OH, Fmoc-Arg(Pbf)-OH, Fmoc-Cys(Trt)-OH, Fmoc-Glu(OtBu)-OH, Fmoc-Glu(O-2-PhiPr)-OH, Fmoc-Gly-OH, Fmoc-Leu-OH, Fmoc-Lys(Boc)-OH, Fmoc-Lys(Mmt)-OH, Fmoc-Pro-OH, Fmoc-Ser(OtBu)-OH, Fmoc-Ser(Trt)-OH, Fmoc-Thr(OtBu)-OH, Fmoc-Thr(Trt)-OH, Fmoc-Cys(Trt)-OH. TentaGel® S AC and S RAM were bought from Rapp-Polymere GmbH and loaded by hand with the appropriate Fmoc-amino acid. Peptides were synthesized with automated solid phase peptide synthesis on an CEM Liberty Blue Automated Microwave Peptide Synthesizer. Automated synthesis of ADPr-peptides was performed on 50 μmol scale. The first amino acid was manually loaded on the resin using 2 eq. Fmoc-AA-OH, 2 eq. DIC and cat. DMAP while shaking over night. Resin was first swollen for 5 minutes in DMF prior to amino acid coupling. Activation was achieved using DIC/Oxyma. Standard coupling was achieved using 5 eq. Fmoc-amino acid as a 0.2 M DMF solution, 5 eq. DIC as a 0.5 M of DIC/DMF solution and 5 eq. Oxyma as a 1M solution in DMF which was buffered by DIPEA (0.1M) at room temperature for 60 minutes. Non-commercially available ribosylated Fmoc-amino acids were coupled using 3 eq. amino acid as a 0.1M solution in DMF, 3 eq. DIC as a 0.5M of DIC/DMF solution and 3 eq. Oxyma as a 1 M Oxyma/DMF solution which was buffered by DIPEA (0.1M) at room temperature for 180 minutes. When a free N-terminal amine was required in the peptide sequence, a Boc protected amino acid was used in the last coupling. When an acetylated N-terminus was required in the peptide sequence, the resin was treated with a solution of 10% Ac_2_O, 20% DIPEA in DMF (8 ml/gr resin) for 2x10 minutes. Standard Fmoc deprotection was achieved by 20% v/v piperidine/DMF at RT for 10 minutes (2 cycles). Synthesis quality could be monitored by UV absorption of dibenzofulvene released during Fmoc deprotection. The quality of the peptide was checked by a test deprotection. A small amount of resin (tip of spatula) was washed 3 times with DCM. 100 μl of cleavage condition 1 or 2 was added, and the resulting mixture was shaken for 1 h at RT. The mixture was filtered into 1 ml of cold Et_2_O and centrifuged at maximum speed for 3 min. The supernatant was discarded, and the pellet dissolved in Magic solution (~1 mg/ml) and analysed by LC-MS. After the last coupling, the resin was washed 3 times with DCM, dried by N_2_ and stored at -20 °C.

General procedure B: on-resin deprotection and phosphorylation.

After peptide elongation, the resin was treated with an 8.8M HF/pyridine solution (10 ml/gr resin) for 30 minutes while shaking. The resin was washed with pyridine and the treatment was repeated once, after which it was extensively washed with pyridine and DCM yielding the desilylated ribosyl peptide intermediate. Hereafter, the resin was washed extensively with MeCN and flushed with N_2_ to remove all traces of water. A solution of (AllO)_2_PN(*i*Pr)_2_ **25** or (FmO)_2_PN(*i*Pr)_2_ **21** (5 eq., 0.25M in MeCN) was added, followed by ETT activator (10 eq., 0.25M in MeCN). The resin was agitated for 30 minutes, followed by washing with MeCN. A solution of CSO (8 ml/gr resin, 0.5M in MeCN) was added and the mixture was shaken for 30 minutes, after which the resin was washed with MeCN and DCM.

General procedure C: deprotection of Fm groups

The Fm groups were cleaved by treatment of the resin with DBU (10% $\frac{v}{v}$ in DMF, 8 ml/gr resin) for 15 minutes followed by washing with DMF. The treatment was repeated after which the resin was washed with DMF and DCM, yielding the phosphoribosylated intermediate.

General procedure D: deprotection of Allyl groups

The All groups were cleaved by treatment of the resin with a solution consisting of freshly prepared Pd(PPh_3_)_4­_ (8.7 mM) and DMBA (147 mM) in DCM (8 ml/gr resin, DCM was purged with N_2_ prior to use) for 15 minutes. The resin was rinsed with DCM after which the treatment was repeated thrice yielding the phosphoribosylated peptide intermediate.

General procedure E: Global deprotection.

The resin was globally deprotected and cleaved from the resin with cleavage cocktail **A** (16 ml/g resin, 50% $\frac{v}{v}$ TFA, 2.5% $\frac{v}{v}$ TIS in DCM) or cleavage cocktail **B** (20 ml/g resin, 5% $\frac{v}{v}$ TFA, 2.5% $\frac{v}{v}$ TIS in DCM) for 1 hour, after which the solution was filtered into cold Et_2_O (5 times the volume of cleavage cocktail). The resin was washed with the respective cleavage cocktail (2 ml/g resin), which was again filtered into the Et_2_O. The precipitated peptide was centrifuged for 5 minutes after which the supernatant was discarded. The resulting pellet was resuspended in Et_2_O and again centrifuged, followed by removal of the supernatant. The crude peptide was dissolved in a NH_4_OAc solution of 1:1 MilliQ:MeCN (100 mM) and lyophilized.

General procedure F: mono-ADPr-peptide phosphate condensation.

DMC·HCl (169 mg, 1.0 mmol, 10 eq. relative to AMP) and imidazole (136 mg, 2.0 mmol, 20 eq. relative to AMP) were dissolved in D_2_O (250 μl) in an Eppendorf tube. D_2_O was added until a total volume of 500 μl, after which the mixture was shaken for 5 minutes. The mixture was added to an Eppendorf containing AMP (39 mg, 0.1 mmol), after which it was shaken for 45 minutes. An Eppendorf tube was loaded with Pr-peptide (1 eq.) and ZnCl_2_ (100 eq.) after which it was dissolved in D_2_O (66 mM relative to Pr-peptide). A solution of the activated AMP-im (5 eq., 200 mM) was added to the Pr-peptide (final concentration of 25mM) and the mixture was shaken for 2.5 hours at 40°C. After 2.5 hours, another freshly prepared portion of activated AMP-Im (5 eq., 200 mM) was added to the condensation reaction. After 5 hours of total reaction time, the mixture was diluted with 5 times the reaction volume of MilliQ and desalted using SEC. The fractions containing the mono-ADPr-peptide were collected, lyophilized repeatedly and purified using HPLC.

Synthesis of phosphoribosyl-peptides.

H2B1C Ser6Pr: Ac-Pro-Ala-Lys-Ser(O^γ^-[5’-O-phosphate-α-D-ribosyl])-Ala-Pro-Ala-Pro-Lys-Lys-Gly-OH (1)

The general procedures **A**-**C** and **E** were followed as described to 50 μmol Tentagel® S AC resin. The amino acids used were Fmoc-Pro-OH, Fmoc-Ala-OH, Fmoc-Lys(Boc)-OH, Fmoc-Gly-OH and Fmoc-Ser(ribosyl)-OH building block **22**^[39]^. On-resin phosphorylation was achieved with (FmO)_2_PN(*i*Pr)_2_ **21**. Global deprotection was achieved using cleavage cocktail **A** for 1 hour. The Pr-peptide was purified by automated column chromatography (C-18 silica, 0% -> 50% H_2_O/ACN +10 mM NH_4_OAc) and compound **1** was obtained as a white solid (9.85 mg, 7.5 μmol, 8%). **^1^H-NMR** (600 MHz, D_2_O) δ 5.11 (d, J = 4.4 Hz, 1H, H-1) **^31^P-NMR** (202 MHz, D_2_O) 3.99. **LC-MS** (00% -> 50% MeCN) Rt = 3.56 min. **LRMS** calculated [M+H]^+^ = 1305.64, [M+2H]^2+^ = 653.32; observed m/z 1305.75, 653.50.

HPF1 Ser97Pr: H-Thr-Lys-Lys-Lys-Ser(O^γ^-[5’-O-phosphate-α-D-ribosyl])-Thr-Gly-Leu-Asn-OH (2)

The general procedures **A**-**C** and **E** were followed as described to 50 μmol Tentagel® S AC resin. The amino acids used were Fmoc-Thr(*t*Bu)-OH, Fmoc-Lys(Boc)-OH, Fmoc-Gly-OH, Fmoc-Leu-OH, Fmoc-Asn(Trt)-OH and ribosyl building block **22**^[39]^. On-resin phosphorylation was achieved with (FmO)_2_PN(*i*Pr)_2_ **21**. Global deprotection was achieved using cleavage cocktail **A** for 1 hour. The Pr-peptide was deemed of sufficient quality to continue crude with the condensation reactions. **LC-MS** (00% -> 50% MeCN) Rt = 0.89 min. **LRMS** calculated [M+H]^+^ = 1188.58, [M+2H]^2+^ = 594.79; observed m/z 1188.67, 595.00.

PARP7 Cys721Pr: Ac-Gln-Glu-Asp-Phe-Cys(S^γ^-[5’-O-phosphate-α-D-ribosyl])-Phe-Leu-Ser-Ser-OH (3)

The general procedures **A**-**C** and **E** were followed as described to 50 μmol Tentagel® S AC resin. The amino acids used were Fmoc-Gln(Trt)-OH, Fmoc-Glu(*t*Bu)-OH, Fmoc-Asp(*t*Bu)-OH, Fmoc-Phe-OH, Fmoc-Leu-OH, Fmoc-Ser(*t*Bu)-OH and ribosyl building block **23**^[39]^. On-resin phosphorylation was achieved with (FmO)_2_PN(*i*Pr)_2_ **21**. Global deprotection was achieved using cleavage cocktail **A** for 1 hour. The Pr-peptide was deemed of sufficient quality to continue crude with the condensation reactions. **LC-MS** (10% -> 50% MeCN) Rt = 0.89 min. LRMS calculated [M+H]^+^ = 1329.45, [M+2H]^2+^ = 665.73; observed m/z 1329.25, 665.25.

CBX4 Tyr207Pr: Ac-Ala-Ala-Lys-Gly-Tyr(O^η^-[5’-O-phosphate-α-D-ribosyl])-Leu-Gly-Ala-Val-OH (4)

The general procedures **A**-**C** and **E** were followed as described to 50 μmol Tentagel® S AC resin. The amino acids used were Fmoc-Ala-OH, Fmoc-Lys(MMt)-OH, Fmoc-Gly-OH, Fmoc-Leu-OH, Fmoc-Val-OH and ribosyl building block **24**^[21]^. On-resin phosphorylation was achieved with (FmO)_2_PN(*i*Pr)_2_ **21**. Global deprotection was achieved using cleavage cocktail **B** for 1 hour. The Pr-peptide was deemed of sufficient quality to continue crude with the condensation reactions. **LC-MS** (10% -> 50% MeCN) Rt = 3.74 min. **LRMS** calculated [M+H]^+^ = 1103.49, [M+2H]^2+^ = 552.75; observed m/z 1103.58, 552.17.

rS1 Arg139Pr: Ac-Val-Arg(N^η^-[5’-O-phosphate-α-D-ribosyl])-Pro-Val-NH_2_ (5)

The general procedure **A** was followed as described to 50 μmol Tentagel® S AC resin. The amino acids used were Fmoc-Val-OH, Fmoc-Pro-OH and Fmoc-Orn(Alloc)-OH. The Alloc on ornithine was deprotected using a solution consisting of freshly prepared Pd(PPh_3_)_4­_ (8.7 mM) and DMBA (147 mM) in DCM (8 ml/gr resin, DCM was purged with N_2_ prior to use) for 15 minutes. The resin was rinsed with DCM after which the treatment was repeated thrice. The resin was washed with DMF, after which the ribosyl building block **33**^[40]^ was coupled. **33** (3 eq., 0.1M in DMF) was added to the resin after which TEA (30 eq.) and AgNO_3_ (3 eq.) were added. The syringe was wrapped in aluminium foil to protect it from light and shaken overnight. The resin was washed with DMF and DCM after which General procedures **B**-**C** and **E** were followed. On-resin phosphorylation was achieved with (FmO)_2_PN(*i*Pr)_2_ **21**. Global deprotection was achieved using cleavage cocktail **A** for 1 hour. The Pr-peptide was deemed of sufficient quality to continue crude with the condensation reactions. **LC-MS** (10% -> 50% MeCN) Rt = 3.42 min. **LRMS** calculated [M+H]^+^ = 723.34; observed m/z 723.42.

Synthesis of ADPr-peptides

HPF1 Ser97ADPr: H-Thr-Lys-Lys-Lys-Ser(O^γ^-[5’-O-adenosine-diphosphate-α-D-ribosyl])-Thr-Gly-Leu-Asn-OH (6)

General procedure **F** was applied to 5 μmol of crude Pr-peptide **2**. The activated AMP-Im cocktail (125 μl, 25 μmol, 5 eq.) was added to a solution of Pr-peptide (5.9 mg, 5 μmol, 1 eq.) and ZnCl_2_ (68 mg, 500 μmol, 100 eq.) in D_2_O (75 μl, 66mM). HPLC purification using an ammonium acetate buffer, followed by repeated lyophilization yielded the titled ADPr-peptide as a white powder (0.77 mg, 0.51 μmol, 10%). **^1^H-NMR** (400 MHz, D_2_O) δ 8.47 (s, 1H, H-2), 8.23 (s, 1H, H-8), 6.10 (d, *J* = 5.9 Hz, 1H, H-1’), 5.01 – 4.97 (m, 1H), H-1”). **^31^P-NMR** (162 MHz, D_2_O) δ -10.44, -10.57, -10.58, -10.71. **LC-MS** (00% -> 20% MeCN) Rt = 4.82 min. **HRMS** [C_56_H_98_N_18_O_27_P_2_+ 2H^+^] found: 759.3234, calculated: 759.3235.

PARP7-Cys721ADPr: Ac-Gln-Glu-Asp-Phe-Cys(Sγ-[5’-O-adenosine-diphosphate-α-D-ribosyl])-Phe-Leu-Ser-Ser-OH (7)

General procedure **F** was applied to 1 μmol of crude Pr-peptide **3**. The activated AMP-Im cocktail (25 μl, 5 μmol, 5 eq.) was added to a solution of Pr-peptide (1.3 mg, 1 μmol, 1 eq.) and ZnCl_2_ (14 mg, 100 μmol, 100 eq.) in D_2_O (15 μl, 66mM). HPLC purification using an ammonium acetate buffer, followed by repeated lyophilization yielded the titled ADPr-peptide as a white powder (0.31 mg, 0.19 μmol, 20%). **^1^H-NMR** (400 MHz, D_2_O) δ 8.45 (s, 1H, H-2), 8.14 (s, 1H, H-8), 7.30 – 7.07 (m, 10H, Phe arom.), 6.04 (d, *J* = 5.7 Hz, 1H, H-1’), 5.38 (d, *J* = 4.6 Hz, 1H, H-1”). **^31^P NMR** (162 MHz, D_2_O) δ -10.29, -10.42, -10.54, -10.66. **LC-MS** (00% -> 50% MeCN) Rt = 5.80 min. **HRMS** [C_64_H_89_N_15_O_31_P_2_S+ 2H^+^] found: 829.7600, calculated: 829.7595.

CBX4 Tyr207ADPr: Ac-Ala-Ala-Lys-Gly-Tyr(O^η^-[5’-O-adenosine-diphosphate-α-D-ribosyl])-Leu-Gly-Ala-Val-OH (8)

General procedure **F** was applied to 5 μmol of crude Pr-peptide **4**. The activated AMP-Im cocktail (125 μl, 25 μmol, 5 eq.) was added to a solution of Pr-peptide (5.5 mg, 5 μmol, 1 eq.) and ZnCl_2_ (68 mg, 500 μmol, 100 eq.) in D_2_O (75 μl, 66mM). HPLC purification using an ammonium acetate buffer, followed by repeated lyophilization yielded the titled ADPr-peptide as a white powder (0.94 mg, 0.66 μmol, 13%). **^1^H NMR** (400 MHz, D_2_O) δ 8.49 (s, 1H, H-2), 8.22 (s, 1H, H-8), 6.99 (d, *J* = 8.4 Hz, 2H, Tyr arom.), 6.84 (d, *J* = 8.2 Hz, 2H Tyr, arom.), 6.06 (d, *J* = 5.6 Hz, 1H, H-1’), 5.51 (d, *J* = 4.5 Hz, 1H, H-1”). **^31^P NMR** (162 MHz, D_2_O) δ -10.36, -10.49, -10.61, -10.75. **LC-MS** (00% -> 50% MeCN) Rt = 4.78 min. **HRMS** [C_56_H_87_N_15_O_25_P_2_+ 2H^+^] found: 716.7808, calculated: 716.7809.

rS1 Arg139ADPr: Ac-Val-Arg(N^η^-[5’-O-adenosine-diphosphate-α-D-ribosyl])-Pro-Val-NH_2_ (9)

General procedure **F** was applied to 1 μmol of crude Pr-peptide **5**. The activated AMP-Im cocktail (25 μl, 5 μmol, 5 eq.) was added to a solution of Pr-peptide (0.72 mg, 1 μmol, 1 eq.) and ZnCl_2_ (14 mg, 100 μmol, 100 eq.) in D_2_O (15 μl, 66mM). HPLC purification using an ammonium acetate buffer, followed by repeated lyophilization yielded the titled ADPr-peptide as a white powder (0.28 mg, 0.28 μmol, 28%). **^1^H NMR** (400 MHz, D_2_O) δ 8.58 – 8.45 (m, 1H, H-2), 8.23 – 8.20 (m, 1H, H-8), 6.09 (d, *J* = 6.0 Hz, 1H, H-1’), 5.29 (d, *J* = 4.4 Hz, 0.4H, H-1” α/β), 5.09 (d, *J* = 5.5 Hz, 0.3H, H-1” α/β), 4.85 (s, 1H). **^31^P NMR** (162 MHz, D_2_O) δ -10.19, -10.22, -10.36, -10.49, -10.59, -10.64, -10.72, -10.77. **LC-MS** (00% -> 50% MeCN) Rt = 3.86 min. **HRMS** [C_38_H_63_N_13_O_18_P_2_+ H^+^] found: 1052.3989, calculated: 1052.3962.

H2B1C Ser6(ADPr)_2_: Ac-Pro-Ala-Lys-Ser(O^γ^-[ADPr_2_])-Ala-Pro-Ala-Pro-Lys-Lys-Gly-OH (10)

DMC-HCl (169 mg, 1.0 mmol) and imidazole (136 mg, 2.0 mmol) were dissolved in D_2_O (250 μl) in an Eppendorf tube. D_2_O was added until a total volume of 500 μl, after which the mixture was shaken for 5 minutes. The mixture was added to an Eppendorf containing phosphoadenosyl ADPr **18** (1.36 μmol, 1.2 eq.) to a concentration of 0.2M of phosphate, after which it was shaken for 45 minutes. An Eppendorf tube was loaded with Pr-peptide **1** (1.5 mg, 1.13 μmol, 1 eq.) and ZnCl_2_ (3.1 mg, 23 μmol, 20 eq.) after which it was dissolved in D_2_O (38 μl, 30 mM relative to Pr-peptide). A solution of the activated phosphoribosyl (7.55 μl, 1.36 μmol, 1.2 eq., 200 mM) was added to the Pr-peptide and the mixture was shaken at 40°C over night. The mixture was diluted with 5 times the reaction volume of MilliQ and desalted using SEC. The fractions containing the titled ADPr-peptide were collected, lyophilized repeatedly. IEX purification using an ammonium acetate buffer gradient of 20 mM to 1.0 M, followed by repeated lyophilization yielded the titled ADPr-peptide as a white powder (0.28 mg, 0.13 μmol, 12%). **^1^H-NMR** (500 MHz, D_2_O) δ 8.36 (s, 1H, H-2), 8.34 (s, 1H ,H-2), 8.12 (s, 1H, H-8), 8.10 (s, 1H, H-8), 6.14 (d, *J* = 3.6 Hz, 1H, H-1’), 5.96 (d, *J* = 6.0 Hz, 1H, H-1’), 5.24 (d, *J* = 4.3 Hz, 1H, H-1”), 4.95 (bs, 1H, H-1”). **^31^P-NMR** (162 MHz, D_2_O) δ -11.14 – -11.61 (m). **LC-MS** (00% -> 20% MeCN) Rt = 5.36 min. **HRMS** [C_79_H_126_N_24_O_40_P_4_+ 2H^+^] found: 1088.3856, calculated: 1088.3830.

Synthesis of phosphoadenosyl-ADPr

**1-*O*-Allyl-2,3-di-*O*-*p*-methoxybenzyl-5-*O*-tert-butyldiphenylsilyl-α,β-d-ribofuranose (40)**

Diol **39** ^[45]^ (2.14 g, 5.00 mmol) was co-evaporated with toluene (1x) and dissolved in a mixture of dry DMF/THF (7/1, 50 mL). PMB-Br (2.2 mL, 15.00 mmol, 3.0 eq) and sodium hydride (60% dispersion in mineral oil, 0.60 g, 15.00 mmol, 3.0 eq.) were slowly added at 0 °C and the reaction mixture was stirred at room temperature overnight. Then, the reaction mixture was quenched with H_2_O, diluted with Et_2_O and the organic phase was washed with H_2_O (2x) and brine (1x). The organic layer was dried over MgSO_4_, filtered and concentrated under reduced pressure. Purification by silica gel column chromatography (10% → 30% Et_2_O in pentane) afforded ether **40** as a yellow oil and as an anomeric mixture (2.96 g, 4.42 mmol, α:β ratio; 0.17:0.83, with an overall yield of 89%). *Note: The α and β anomers were partially separated by silica gel column chromatography purification for clean NMR assignment.* Data for α anomer: **^1^H NMR (300 MHz, CDCl_3_)** δ 7.62 – 7.53 (m, 4H, arom.), 7.43 – 7.32 (m, 6H, arom.), 7.30 – 7.28 (m, 2H, arom.), 7.25 – 7.21 (m, 2H, arom.), 6.90 – 6.86 (m, 2H, arom.), 6.83 – 6.79 (m, 2H, arom.), 5.98 (ddt, *J* = 17.1, 11.1, 6.5, 4.8 Hz, 1H, CH_2_*CH*CH_2_), 5.37 – 5.17 (m, 2H, CH_2_CH*CH_2_*), 5.03 (d, *J* = 4.3 Hz, 1H, H1), 4.61 – 4.58 (m, 3H, CH_2_ PMB), 4.49 (d, *J* = 12.3 Hz, 1H, CH_2_ PMB), 4.33 – 4.25 (m, 1H, O*CH_2_*CH), 4.17 – 4.10 (m, 2H, H4, O*CH_2_*CH), 3.95 (dd, *J* = 6.5, 3.0 Hz, 1H, H3), 3.80 – 3.78 (m, 4H, H2, CH_3_ PMB), 3.78 (s, 3H, CH_3_ PMB), 3.61 (AB, *J* = 11.1, 3.6 Hz, 1H, H5), 3.51 (AB, *J* = 11.1, 3.3 Hz, 1H, H5), 0.95 (s, 9H, CH_3_ TBDPS). **^13^C NMR (75 MHz, CDCl_3_)** δ 159.4, 159.2 (Cq. arom.), 135.8 (arom.), 135.0 (CH_2_*C*HCH_2_), 133.3, 130.6, 130.2 (Cq. arom.), 129.9, 129.7, 127.8 (arom.), 117.3 (CH_2_CH*C*H_2_), 113.9, 113.8 (arom.), 100.3 (C1), 83.5 (C4), 77.8 (C2), 74.9 (C3), 72.2, 72.0 (CH_2_ PMB), 68.7 (O*C*H_2_CH), 64.3 (C5), 55.4, 55.4 (CH_3_ PMB), 26.9 (CH_3_ TBDPS), 19.3 (Cq. TBDPS). Data for β anomer: **^1^H NMR (300 MHz, CDCl_3_)** δ 7.70 – 7.64 (m, 4H, arom.), 7.44 – 7.32 (m, 6H, arom.), 7.30 – 7.25 (m, 2H, arom.), 7.19 (d, *J* = 8.6 Hz, 2H, arom.), 6.86 (d, *J* = 8.6 Hz, 2H, arom.), 6.81 (d, *J* = 8.6 Hz, 2H, arom.), 5.81 (ddt, *J* = 16.7, 10.9, 5.7 Hz, 1H, CH_2_*CH*CH_2_), 5.23 – 5.10 (m, 2H, CH_2_CH*CH_2_*), 5.05 (s, 1H, H1), 4.60 (d, *J* = 11.7 Hz, 1H, CH_2_ PMB), 4.54 (d, *J* = 11.7 Hz, 1H, CH_2_ PMB), 4.46 (d, *J* = 11.4 Hz, 1H, CH_2_ PMB), 4.38 (d, *J* = 11.4 Hz, 1H, CH_2_ PMB), 4.27 – 4.21 (m, 1H, H4), 4.19 – 4.10 (m, 2H, H3, O*CH_2_*CH), 3.93 (dd, *J* = 12.9, 6.2 Hz, 1H, O*CH_2_*CH), 3.87 (d, *J* = 4.8 Hz, 1H, H2), 3.82 (d, *J* = 3.9 Hz, 1H, H5), 3.80 (s, 3H, CH_3_ PMB), 3.78 (s, 3H, CH_3_ PMB), 3.69 (AB, *J* = 11.1, 4.6 Hz, 1H, H5), 1.03 (s, 9H, CH_3_ TBDPS). **^13^C NMR (75 MHz, CDCl_3_)** δ 159.5, 159.4 (Cq. arom.), 135.8 (arom.), 134.3 (CH_2_*C*HCH_2_), 133.6, 130.2, 130.2 (Cq. arom.), 129.8, 129.5, 127.8 (arom.), 117.3 (CH_2_CH*C*H_2_), 113.9, 113.9 (arom.), 104.6 (C1), 82.2 (C4), 79.8 (C2), 77.6 (C3), 72.1, 72.1 (CH_2_ PMB), 68.6 (O*C*H_2_CH), 64.6 (C5), 55.4, 55.4 (CH_3_ PMB), 27.0 (CH_3_ TBDPS), 19.4 (Cq. TBDPS). Spectral data were in accordance with those reported in the literature. ^[45]^

**2,3-Di-*O*-*p*-methoxybenzyl-5-*O*-tert-butyldiphenylsilyl****-α,β-d-ribofuranose (41)**

[Ir(COD)(PMePh_2_)_2_]PF_6_ (93 mg, 0.11 mmol, 0.01 eq.) was added to a flame-dried flask and dissolved in dry THF (11 mL). The resulting solution was purged with Ar, followed with H_2_ until the solution became clear and then again with Ar. Allyl riboside **40** (7.37 g, 11.0 mmol, 1.0 eq) was co-evaporated with toluene (3x), dissolved in dry THF (55 mL) and slowly added to the flask containing the iridium catalyst. The resulting mixture was stirred at room temperature for 2 days. After complete conversion into the vinyl ether, sat. aq. NaHCO_3_ (66 mL) and I_2_ (3.35 g, 13.2 mmol, 1.2 eq) were added and the reaction mixture was stirred for 30 minutes. Then, the reaction was quenched with sat. aq. Na_2_S_2_O_3_ and the aqueous phase was extracted with EtOAc (1x). The organic layer was dried over MgSO_4_, filtered and concentrated under reduced pressure. Purification by silica gel column chromatography (20% → 50% Et_2_O in pentane) afforded hemiacetal **41** as a yellow oil and as an anomeric mixture (5.95 g, 9.46 mmol, α:β ratio; 0.74:0.26, with an overall yield of 86%). Data for anomeric mixture: **^1^H NMR (400 MHz, CDCl_3_)** δ 7.64 – 7.58 (m, 4H, arom.), 7.44 – 7.35 (m, 6H, arom.), 7.32 – 7.17 (m, 4H, arom.), 6.89 – 6.82 (m, 4H, arom.), 5.30 – 5.25 (m, 1H, H1-α, H1-β), 4.66 – 4.45 (m, 4H, CH_2_ PMB), 4.29 – 4.25 (m, 1.26H, H4-α, H3-β, H4-β), 4.17 (d, *J* = 11.3 Hz, 0.74H, OH-α), 4.05 (dd, *J* = 4.9, 1.7 Hz, 0.74H, H3-α), 3.98 – 3.95 (m, 0.74H, H2-α), 3.86 – 3.83 (m, 0.52H, H2-β, H5-β), 3.80 – 3.78 (m, 6.26H, CH_3_ PMB, H5-β), 3.62 – 3.58 (m, 1.48H, H5-α), 3.11 (d, *J* = 6.5 Hz, 0.26H, OH-β), 1.01 (s, 2.3H, CH_3_ TBDPS-β), 0.99 (s, 6.7H, CH_3_ TBDPS-α). **^13^C NMR (101 MHz, CDCl_3_)** δ 159.5, 159.5 (Cq. arom.), 135.8, 135.7, 135.7, 135.6 (arom.), 133.2, 133.0, 132.7 (Cq. arom.), 130.1, 130.0, 130.0, 129.9, 129.9 (arom.), 129.8, 129.7 (Cq. arom.), 129.7, 129.6, 129.6, 128.0, 127.9, 127.9, 114.0, 114.0, 113.9 (arom.), 100.2 (C1-β), 96.4 (C1-α), 82.6 (C4-α), 82.3 (C4-β), 80.2 (C2-β), 77.8 (C3-β), 77.5 (C2-α), 76.1 (C3-α), 72.6, 72.1, 72.1, 72.0 (CH_2_ PMB), 64.1 (C5-β), 63.3 (C5-α), 55.4 (CH_3_ PMB), 26.9 (CH_3_ TBDPS), 19.3 (Cq. TBDPS). Spectral data were in accordance with those reported in the literature. ^[45]^

**1-*O*-((*N*-Phenyl)-2,2,2-trifluoroacetimido)-2,3-di-*O*-*p*-methoxybenzyl-5-*O*-tert-butyldiphenylsilyl****-α,β-d-ribofuranose (42)**

Hemiacetal **41** (5.47 g, 8.71 mmol) was dissolved in acetone (44 mL). Cs_2_CO_3_ (4.26 g, 13.07 mmol, 1.5 eq) and PTFAI-Cl (1.55 mL, 9.58 mmol, 1.1 eq) were added and the reaction mixture was stirred at room temperature for 3 hours. Then, the reaction mixture was filtered over Celite and concentrated under reduced pressure. Purification by silica gel column chromatography neutralized with 1% Et_3_N (5% → 20% Et_2_O in pentane) afforded imidate **42** as a yellow oil and as an anomeric mixture (6.43 g, 8.00 mmol, α:β ratio; 0.45:0.55, with an overall yield of 92%). Data for anomeric mixture: **^1^H NMR (400 MHz, CDCl_3_)** δ 7.71 – 7.64 (m, 2H, arom.), 7.59 – 7.55 (m, 2H, arom.), 7.42 – 7.34 (m, 7H, arom.), 7.28 – 7.22 (m, 5H, arom.), 7.11 – 7.06 (m, 1H, arom.), 6.86 – 6.80 (m, 6H, arom.), 6.43 (bs, 0.45H, H1-α), 6.27 (bs, 0.55H, H1-β), 4.63 (d, *J* = 13.2 Hz, 2H, CH_2_ PMB), 4.56 – 4.38 (m, 2H, CH_2_ PMB), 4.37 – 4.29 (m, 1H, H4-α, H4-β), 4.26 – 4.16 (m, 0.55H, H3-β), 4.13 – 4.04 (m, 1.45H, H2-α, H2-β, H3-α), 3.89 (d, *J* = 9.2 Hz, 0.55H, H5-β), 3.79 – 3.77 (m, 6.55H, CH_3_ PMB, H5-β), 3.67 (d, *J* = 10.6 Hz, 0.45H, H5-α), 3.56 (d, *J* = 10.3 Hz, 0.45H, H5-α), 1.04 (s, 4.95H, CH_3_ TBDPS-β), 0.96 (s, 4.05H, CH_3_ TBDPS-α). **^13^C NMR (101 MHz, CDCl_3_)** δ 159.6, 159.5, 159.3, 144.0 (Cq. arom.), 135.8, 135.7, 135.7, 135.7 (arom.), 133.5, 133.2, 133.1, 132.8, 130.5 (Cq. arom.), 130.0, 130.0, 130.0, 129.8, 129.8 (arom.), 129.8 (Cq. arom.), 129.6, 129.5, 129.4, 128.8, 128.8, 127.9, 127.9, 127.8, 127.8, 126.5, 124.3, 120.6, 119.7, 114.0, 114.0, 113.9, 113.8 (arom.), 102.9 (C1-β), 85.8 (C4-α), 83.5 (C4-β), 78.8 (C2-α), 78.5 (C2-β), 76.1 (C3-β), 75.4 (C3-α), 73.0, 72.4, 72.3, 72.0 (CH_2_ PMB), 63.8 (C5-α), 63.4 (C5-β), 55.4, 55.3 (CH_3_ PMB), 26.9, 26.8 (CH_3_ TBDPS), 19.3, 19.3 (Cq. TBDPS). Spectral data were in accordance with those reported in the literature. ^[45]^

**α-1,3,5-Tri-*O*-benzoyl-2’,3’-di-*O*-*p*-methoxybenzyl-5’-*O*-tert-butyldiphenylsilyl-parobiose (44)**

1,3,5-Tri-*O*-benzoyl-α-d-ribofuranose **43** (0.97 g, 2.10 mmol) and imidate **42** (1.84 g, 2.30 mmol, 1.1 eq) were co-evaporated with toluene (1x), 1,4-dioxane (2x) and DCE (1x) and dissolved in dry DCM (42 mL). Freshly activated 3Å molecular sieves were added and the resulting mixture was stirred at room temperature for 1 hour. Next, the mixture was cooled to -78 °C, TMSOTf (8.3 µL, 46 µmol, 0.02 eq) was added and the reaction mixture was stirred at the same temperature for 10 minutes. The reaction was carefully quenched with Et_3_N (3.2 mL) and concentrated under reduced pressure. Purification by silica gel column chromatography (20% → 50% Et_2_O in pentane) afforded disaccharide **44** as a white foam (1.89 g, 1.76 mmol, 77%). **^1^H NMR (400 MHz, CDCl_3_)** δ 8.15 (ddt, *J* = 7.8, 2.6, 1.2 Hz, 4H, arom.), 8.06 – 8.03 (m, 2H, arom.), 7.58 – 7.48 (m, 7H, arom.), 7.40 – 7.28 (m, 10H, arom.), 7.24 – 7.20 (m, 2H, arom.), 7.04 – 6.99 (m, 4H, arom.), 6.80 (d, *J* = 4.2 Hz, 1H, H1), 6.68 – 6.62 (m, 4H, arom.), 5.64 (dd, *J* = 6.3, 1.9 Hz, 1H, H3), 5.26 (d, *J* = 4.2 Hz, 1H, H1’), 4.76 – 4.73 (m, 1H, H4), 4.71 (dd, *J* = 6.4, 4.3 Hz, 1H, H2), 4.65 (AB, *J* = 12.0, 3.4 Hz, 1H, H5), 4.58 (AB, *J* = 12.0, 4.3 Hz, 1H, H5), 4.50 (d, *J* = 11.7 Hz, 1H, CH_2_ PMB), 4.36 (d, *J* = 11.7 Hz, 2H, CH_2_ PMB), 4.27 (d, *J* = 11.7 Hz, 1H, CH_2_ PMB), 4.12 (q, *J* = 3.2 Hz, 1H, H4’), 3.97 (dd, *J* = 6.2, 3.8 Hz, 1H, H3’), 3.90 (dd, *J* = 6.2, 4.2 Hz, 1H, H2’), 3.74 (s, 3H, CH_3_ PMB), 3.72 (s, 3H, CH_3_ PMB), 3.60 (AB, *J* = 11.3, 3.4 Hz, 1H, H5’), 3.50 (AB, *J* = 11.4, 2.8 Hz, 1H, H5’), 0.93 (s, 9H, CH_3_ TBDPS). **^13^C NMR (101 MHz, CDCl_3_)** δ 166.5, 166.2, 165.9 (CO Bz), 159.1, 159.0 (Cq. arom.), 135.7, 135.6, 133.3, 133.3, 133.2 (arom.), 133.1, 130.6 (Cq. arom.), 130.3 (arom.), 130.3, 130.2, 130.0 (Cq. arom.), 129.9, 129.9, 129.8 (arom.), 129.8 (Cq. arom.), 129.3, 129.1, 128.6, 128.6, 128.4, 128.4, 127.8, 127.8, 113.7 (arom.), 102.0 (C1’), 95.3 (C1), 83.6 (C4’), 83.4 (C4), 78.4 (C2’), 75.5 (C3’), 75.2 (C2), 72.4 (C3), 72.1, 72.0 (CH_2_ PMB), 64.5 (C5), 63.7 (C5’), 55.3, 55.3 (CH_3_ PMB), 26.9 (CH_3_ TBDPS), 19.3 (Cq. TBDPS).

**α-1,3,5-Tri-*O*-benzoyl-5’-*O*-tert-butyldiphenylsilyl-parobiose (45)**

Compound **44** (2.17 g, 2.33 mmol) was dissolved in dry DCM (23 mL). TFA (1.2 mL, 15.13 mmol, 6.5 eq) was added and the reaction mixture was stirred at room temperature for 1 hour. Then, the reaction was carefully quenched with sat. aq. NaHCO_3_ and the aqueous phase was extracted with DCM (3x). The combined organic layers were dried over MgSO_4_, filtered and concentrated under reduced pressure. Purification by silica gel column chromatography (40% Et_2_O in pentane) afforded diol **45** as a white foam (1.53 g, 1.83 mmol, 79%). **^1^H NMR (400 MHz, CDCl_3_)** δ 8.15 – 8.01 (m, 6H, arom.), 7.66 – 7.52 (m, 7H, arom.), 7.48 – 7.29 (m, 12H, arom.), 6.78 (d, *J* = 4.2 Hz, 1H, H1), 5.73 (dd, *J* = 6.3, 1.9 Hz, 1H, H3), 5.20 (d, *J* = 4.2 Hz, 1H, H1’), 4.89 – 4.85 (m, 1H, H4), 4.77 (dd, *J* = 6.3, 4.3 Hz, 1H, H2), 4.67 (AB, J = 12.1, 3.5 Hz, 1H, H5), 4.60 (AB, J = 12.2, 3.9 Hz, 1H, H5), 4.23 – 4.10 (m, 1H, H2’), 4.10 – 4.00 (m, 2H, H3’, H4’), 3.70 – 3.61 (m, 2H, H5’), 2.66 (d, *J* = 10.3 Hz, 1H, 2’-OH), 2.53 (d, *J* = 9.6 Hz, 1H, 3’-OH), 1.00 (s, 9H, CH_3_ TBDPS). **^13^C NMR (101 MHz, CDCl_3_)** δ 166.9, 166.1, 165.9 (CO Bz), 135.7, 135.6, 133.9, 133.6, 133.5 (arom.), 133.0, 132.9 (Cq. arom.), 130.1, 130.0, 129.9, 129.8 (arom.), 129.5, 129.0 (Cq. arom.), 128.7, 128.7, 128.6, 127.9, 127.9 (arom.), 102.5 (C1’), 95.2 (C1), 87.0 (C4’), 82.8 (C4), 75.2 (C2), 72.7 (C2’), 72.5 (C3), 71.1 (C3’), 64.3 (C5), 63.9 (C5’), 26.9 (CH_3_ TBDPS), 19.3 (Cq. TBDPS).

**α-1,3,5-Tri-*O*-benzoyl-2’,3’-di-*O*-acetyl-5’-*O*-tert-butyldiphenylsilyl-parobiose (46)**

Diol **45** (2.39 g, 1.99 mmol) was co-evaporated with toluene (2x) and dissolved in dry pyridine (13 mL). DMAP (24 mg, 0.20 mmol, 0.1 eq) and Ac_2_O (1.9 mL, 19.91 mmol, 10.0 eq) were added and the reaction mixture was stirred at room temperature for 3.5 hours. Then, the reaction mixture was concentrated under reduced pressure and co-evaporated with toluene (3x). The resulting residue was diluted with EtOAc and the organic phase was washed with sat. aq. NaHCO_3_ (2x) and brine (1x). The organic layer was dried over MgSO_4_, filtered and concentrated under reduced pressure. Purification by silica gel column chromatography (40% Et_2_O in pentane) afforded title compound **46** as a white foam (1.82 g, 1.99 mmol, quantitative). **^1^H NMR (400 MHz, CDCl_3_)** δ 8.16 (ddt, *J* = 9.4, 8.1, 1.1 Hz, 4H, arom.), 8.09 – 8.05 (m, 2H, arom.), 7.67 – 7.50 (m, 7H, arom.), 7.48 – 7.29 (m, 12H, arom.), 6.82 (d, *J* = 4.2 Hz, 1H, H1), 5.75 (dd, *J* = 6.4, 1.7 Hz, 1H, H3), 5.46 (d, *J* = 4.6 Hz, 1H, H1’), 5.38 (dd, *J* = 7.0, 2.5 Hz, 1H, H3’), 4.99 (dd, *J* = 7.0, 4.5 Hz, 1H, H2’), 4.82 (td, *J* = 3.7, 1.6 Hz, 1H, H4), 4.73 – 4.57 (m, 3H, H2, H5), 4.10 (q, *J* = 2.8 Hz, 1H, H4’), 3.74 – 3.62 (m, 2H, H5’), 1.62 (s, 3H, CH_3_ Ac), 1.45 (s, 3H, CH_3_ Ac), 1.01 (s, 9H, CH_3_ TBDPS). **^13^C NMR (101 MHz, CDCl_3_)** δ 170.3, 169.7 (CO Ac), 166.2, 165.9, 165.1 (CO Bz), 135.7, 135.7, 133.5, 133.5 (arom.), 133.0, 132.9 (Cq. arom), 130.2, 130.1 (arom.), 130.0 (Cq. arom.), 129.9, 129.9, 129.8 (arom.), 129.6 (Cq. arom.), 128.7, 128.5, 128.4, 127.9, 127.9 (arom.), 101.1 (C1’), 95.2 (C1), 83.5 (C4), 83.3 (C4’), 75.9 (C2), 71.7 (C3), 71.5 (C2’), 70.0 (C3’), 64.4 (C5), 63.5 (C5’), 26.8 (CH_3_ TBDPS), 19.8 (CH_3_ Ac), 19.3 (Cq. TBDPS).

***N^6^*-Benzoyl-9-(3’,5’-di-*O*-benzoyl-2’’,3’’-*O*-di-acetyl-5’’-*O*-tert-butyldiphenylsilyl-β-parobiosyl)adenine (48)**

Protected disaccharide **46** (3.79 g, 4.13 mmol) and *N*^6^-benzoyladenine **47** (1.98 g, 8.26 mmol, 2.0 eq) were co-evaporated with 1,4-dioxane (2x) and ACN (1x) and dissolved in dry ACN (69 mL). BSTFA (15.4 mL, 57.82 mmol, 14.0 eq) was added and the resulting mixture was stirred at room temperature for 30 minutes. Next, HClO_4_-SiO_2_ (20.65 g, 8.26 mmol, 2.0 eq) was added and the reaction mixture was refluxed overnight. Then, the reaction was carefully quenched with sat. aq. NaHCO_3_ and filtered over Celite. The aqueous phase was extracted with EtOAc (3x) and the combined organic layers were dried over MgSO_4_, filtered and concentrated under reduced pressure. Purification by silica gel column chromatography (20% → 25% acetone in pentane) afforded nucleoside **48** as a white foam (2.80 g, 2.71 mmol, 66%). **^1^H NMR (400 MHz, CDCl_3_)** δ 9.00 (s, 1H, NH), 8.69 (s, 1H, H2), 8.17 (s, 1H, H8), 8.10 (dd, *J* = 10.6, 7.9 Hz, 4H, arom.), 8.02 (d, *J* = 7.4 Hz, 2H, arom.), 7.61 – 7.51 (m, 9H, arom.), 7.46 – 7.33 (m, 10H, arom.), 6.27 (d, *J* = 5.5 Hz, 1H, H1’), 5.92 (t, *J* = 4.5 Hz, 1H, H3’), 5.51 (t, *J* = 5.5 Hz, 1H, H2’), 5.39 – 5.32 (m, 2H, H1’’, H3’’), 4.91 – 4.82 (m, 2H, H2’’, H5’), 4.73 – 4.65 (m, 2H, H4’, H5’), 3.99 (q, *J* = 2.6 Hz, 1H, H4’’), 3.60 (AB, *J* = 11.3, 2.4 Hz, 1H, H5’’), 3.50 (AB, *J* = 11.3, 3.0 Hz, 1H, H5’’), 1.87 (s, 3H, CH_3_ Ac), 1.77 (s, 3H, CH_3_ Ac), 0.98 (s, 9H, CH_3_ TBDPS). **^13^C NMR (101 MHz, CDCl_3_)** δ 170.3, 169.7 (CO Ac), 166.3, 165.4, 164.6 (CO Bz), 153.1 (arom.), 151.8, 149.8 (Cq. arom.), 135.7, 135.7, 133.8 (arom.), 133.7 (Cq. arom.), 133.6, 133.0 (arom.), 133.0, 132.8 (Cq. arom.), 130.0, 130.0, 129.9, 129.9 (arom.), 129.5, 129.4 (Cq. arom.), 129.1, 128.8, 128.7, 128.0, 127.9, 127.9 (arom.), 123.8 (Cq. arom.), 101.7 (C1’’), 87.8 (C1’), 83.2 (C4’’), 81.0 (C4’), 77.9 (C2’), 72.5 (C3’), 71.7 (C2’’), 70.1 (C3’’), 63.7 (C5’), 63.3 (C5’’), 26.8 (CH_3_ TBDPS), 20.4, 20.4 (CH_3_ Ac), 19.3 (Cq. TBDPS).

***N^6^*-Benzoyl-9-(5’’-*O*-tert-butyldiphenylsilyl-β-parobiosyl)adenine (49)**

Nucleoside **48** (4.12 g, 3.99 mmol) was dissolved in a mixture of pyridine/EtOH (2/1, 40 mL). Aq. NaOH (1 M, 24 mL) was slowly added at 0 °C and the reaction mixture was stirred at the same temperature for 1 hour. Then, the reaction mixture was quenched with Amberlite-H^+^ until pH 6, filtered and concentrated under reduced pressure. Purification by silica gel column chromatography (0% → 7.5% MeOH in DCM) afforded tetraol **49** as a white foam (2.73 g, 3.67 mmol, 92%). **^1^H NMR (400 MHz, CDCl_3_)** δ 9.53 (s, 1H, NH), 8.73 (s, 1H, H2), 8.27 (s, 1H, H8), 7.98 (d, *J* = 7.4 Hz, 2H, arom.), 7.59 – 7.51 (m, 5H, arom.), 7.43 – 7.33 (m, 8H, arom.), 6.10 (d, *J* = 7.0 Hz, 1H, H1’), 5.06 (d, *J* = 3.2 Hz, 1H, H1’’), 4.98 (dd, *J* = 6.8, 4.8 Hz, 1H, H2’), 4.58 (d, *J* = 4.6 Hz, 1H, H3’), 4.31 – 4.23 (m, 4H, H4’, H2’’, H3’’, H4’’), 3.93 (d, *J* = 12.1 Hz, 1H, H5’), 3.75 (d, *J* = 12.1 Hz, 1H, H5’), 3.64 (d, *J* = 2.4 Hz, 2H, H5’’), 0.97 (s, 9H, CH_3_ TBDPS). **^13^C NMR (101 MHz, CDCl_3_)** δ 165.2 (CO Bz), 152.3 (C2), 150.8, 150.2 (Cq. arom.), 143.6 (C8), 135.7, 135.6 (arom.), 133.4 (Cq. arom.), 133.1 (arom.), 132.9, 132.7 (Cq. arom.), 130.0, 128.9, 128.2, 128.0 (arom.), 124.2 (Cq. arom.), 101.8 (C1’’), 89.2 (C1’), 87.9 (C4’), 86.4 (C4’’), 79.5 (C2’), 73.4 (C2’’/C3’’), 72.8 (C3’), 71.9 (C2’’/C3’’), 64.2 (C5’’), 63.1 (C5’), 26.9 (CH_3_ TBDPS), 19.3 (Cq. TBDPS).

N6-Benzoyl-9-(3’,2”,3”-tri-O-acetyl-5’-O-dimethoxytrityl-5”-O-tert-butyldiphenylsilyl-β-parobiosyl)adenine (11)

Tetraol **49** (1.77 g, 2.38 mmol) was co-evaporated with pyridine (1x) and dissolved in dry pyridine (12 mL). DMTCl (0.97 g, 2.86 mmol, 1.2 eq) was added and the reaction mixture was stirred at room temperature for 2 hours. After complete conversion into the dimethoxytrityl ether, Ac_2_O (1.4 mL, 14.28 mmol, 6.0 eq) was added at 0 °C and the reaction mixture was stirred at the same temperature for 5 hours. Then, the reaction was carefully quenched with sat. aq. NaHCO_3_ and the aqueous phase was extracted with DCM (3x). The combined organic layers were dried over MgSO_4_, filtered and concentrated under reduced pressure. Purification by silica gel column chromatography (0% → 30% acetone in pentane) afforded title compound **11** as a white foam (2.52 g, 2.15 mmol, 90% over 2 steps). **^1^H NMR (400 MHz, CDCl_3_)** δ 9.14 (s, 1H, NH), 8.76 (s, 1H, H2), 8.16 (s, 1H, H8), 8.04 – 8.02 (m, 2H, arom.), 7.65 – 7.61 (m, 5H, arom.), 7.55 – 7.52 (m, 2H, arom.), 7.42 – 7.30 (m, 14H, arom.), 7.23 – 7.21 (m, 1H, arom.), 6.82 – 6.79 (m, 4H, DMT), 6.27 (d, *J* = 5.9 Hz, 1H, H1’), 5.58 (dd, *J* = 5.0, 3.8 Hz, 1H, H3’), 5.45 (dd, *J* = 7.0, 2.8 Hz, 1H, H3’’), 5.37 (d, *J* = 4.6 Hz, 1H, H1’’), 5.21 (t, *J* = 5.5 Hz, 1H, H2’), 4.95 (dd, *J* = 7.0, 4.6 Hz, 1H, H2’’), 4.34 (q, *J* = 3.4 Hz, 1H, H4’), 4.14 (q, *J* = 2.7 Hz, 1H, H4’’), 3.78 – 3.75 (m, 8H, CH_3_ DMT, H5’’), 3.55 (AB, *J* = 10.6, 3.1 Hz, 1H, H5’), 3.47 (AB, *J* = 10.7, 3.6 Hz, 1H, H5’), 2.11 (s, 3H, CH_3_ Ac), 2.10 (s, 3H, CH_3_ Ac), 1.85 (s, 3H, CH_3_ Ac), 1.02 (s, 9H, CH_3_ TBDPS). **^13^C NMR (101 MHz, CDCl_3_)** δ 170.4, 169.8, 169.7 (CO Ac), 164.7 (CO Bz), 158.8 (Cq. arom.), 153.1 (C2), 151.9, 149.7, 144.4 (Cq. arom.), 141.4 (C8), 135.7, 135.7 (arom.), 135.4, 133.8 (Cq. arom.), 132.9 (arom.), 132.7 (Cq. arom.), 130.2, 130.0, 129.9, 129.0, 128.3, 128.1, 128.0, 127.9, 127.9, 127.3 (arom.), 123.3 (Cq. arom.), 113.4 (arom.), 101.6 (C1’’), 87.1 (Cq. DMT), 86.5 (C1’), 83.2 (C4’’), 82.7 (C4’), 78.6 (C2’), 72.4 (C3’), 71.6 (C2’’), 70.3 (C3’’), 63.5 (C5’’), 63.0 (C5’), 55.3 (CH_3_ DMT), 26.8 (CH_3_ TBDPS), 21.0, 20.4 (CH_3_ Ac), 19.3 (Cq. TBDPS).

*N^6^*-Benzoyl-9-(3’,2”,3”-tri-*O*-acetyl-5”-*O*-tert-butyldiphenylsilyl-β-parobiosyl)adenine (12)

Compound **11** (2.03 g, 1.74 mmol) was dissolved in DCM (17 mL). TFA (270 µL, 3.48 mmol, 2.0 eq) was added and the reaction mixture was stirred at room temperature for 10 minutes. Then, the reaction was carefully quenched with sat. aq. NaHCO_3_ and the aqueous phase was extracted with DCM (3x). The combined organic layers were dried over MgSO_4_, filtered and concentrated under reduced pressure. Purification by silica gel column chromatography (0% → 30% acetone in DCM) afforded alcohol **12** as a white foam (0.94 g, 1.09 mmol, 63%). **^1^H NMR (400 MHz, CDCl_3_)** δ 9.10 (s, 1H, NH), 8.82 (s, 1H, H2), 8.11 (s, 1H, H8), 8.06 – 7.98 (m, 2H, arom.), 7.65 – 7.49 (m, 7H, arom.), 7.46 – 7.30 (m, 6H, arom.), 6.11 (dd, *J* = 11.6, 2.5 Hz, 1H, OH), 6.02 (d, *J* = 7.9 Hz, 1H, H1’), 5.68 (d, *J* = 5.4 Hz, 1H, H3’), 5.37 (dd, *J* = 7.0, 2.9 Hz, 1H, H3’’), 5.14 (dd, *J* = 7.8, 5.3 Hz, 1H, H2’), 5.09 (d, *J* = 4.8 Hz, 1H, H1’’), 4.92 (dd, *J* = 7.0, 4.7 Hz, 1H, H2’’), 4.32 (q, *J* = 1.5 Hz, 1H, H4’), 4.04 – 3.97 (m, 2H, H4’’, H5’), 3.87 (t, *J* = 11.5 Hz, 1H, H5’), 3.70 (AB, J = 2.6 Hz, 1H, H5’’), 3.68 (AB, J = 3.0 Hz, 1H, H5’’), 2.15 (s, 3H, CH_3_ Ac), 2.13 (s, 3H, CH_3_ Ac), 1.97 (s, 3H, CH_3_ Ac), 0.99 (s, 9H, CH_3_ TBDPS). **^13^C NMR (101 MHz, CDCl_3_)** δ 170.2, 169.6, 169.5 (CO Ac), 164.5 (CO Bz), 152.4 (C2), 150.6, 150.5 (Cq. arom.), 143.1 (C8), 135.7 (arom.), 133.5 (Cq. arom.), 133.1 (arom.), 132.9, 132.7 (Cq. arom.), 130.0, 129.9, 129.1, 128.0, 127.9, 127.9 (arom.), 124.6 (Cq. arom.), 101.2 (C1’’), 89.8 (C1’), 86.9 (C4’), 83.1 (C4’’), 77.6 (C2’), 73.8 (C3’), 71.6 (C2’’), 70.2 (C3’’), 63.4 (C5’’), 62.9 (C5’), 26.8 (CH_3_ TBDPS), 21.0, 20.9, 20.4 (CH_3_ Ac), 19.2 (Cq. TBDPS).

*N^6^*-Benzoyl-9-(3’,2”,3”-tri-*O*-acetyl-5’-*O*-(di-fluorenylphosphoryl)-5”-*O*-tert-butyldiphenylsilyl-β-parobiosyl)adenine (13)

Alcohol **12** (1.07 g, 1.23 mmol) was co-evaporated with ACN (3x). Freshly activated 3Å molecular sieves, DCI (0.25 M in dry ACN, 9.8 mL, 2.46 mmol, 2.0 eq) and difluorenylmethyl *N*,*N*-diisopropylphosphoramidite **21** ^[43]^ (0.2 M in dry ACN, 9.2 mL, 1.85 mmol, 1.5 eq) were added and the reaction mixture was stirred at room temperature for 10 minutes. After complete conversion into the phosphite triester indicated by ^31^P-NMR, *t*BuOOH (5.5 M in decane, 2.2 mL, 12.30 mmol, 10.0 eq) was added at 0 °C and the reaction mixture was stirred at the same temperature for 30 minutes. After complete conversion into the phosphotriester, the reaction was carefully quenched with sat. aq. NaHCO_3_ and filtered. Then, the resulting mixture was diluted with EtOAc and the organic phase was washed with H_2_O (1x) and brine (2x). The organic layer was dried over MgSO_4_, filtered and concentrated under reduced pressure. Purification by silica gel column chromatography (0% → 25% acetone in DCM) afforded phosphotriester **13** as a white foam (1.48 g, 1.13 mmol, 92% over 2 steps). **^1^H NMR (400 MHz, CDCl_3_)** δ 8.91 (s, 1H, NH), 8.78 (s, 1H, H2), 8.23 (s, 1H, H8), 8.01 – 7.95 (m, 2H, arom.), 7.71 – 7.15 (m, 29H, arom.), 6.18 (d, *J* = 5.4 Hz, 1H, H1’), 5.44 – 5.38 (m, 2H, H3’, H3’’), 5.28 (d, *J* = 4.6 Hz, 1H, H1’’), 4.98 (t, *J* = 5.4 Hz, 1H, H2’), 4.92 (dd, *J* = 7.0, 4.6 Hz, 1H, H2’’), 4.33 – 4.23 (m, 5H, H4’, CH_2_ Fm), 4.15 – 4.05 (m, 5H, H5’, H4’’, CH Fm), 3.73 (AB, *J* = 11.3, 2.7 Hz, 1H, H5’’), 3.66 (AB, *J* = 11.3, 3.3 Hz, 1H, H5’’), 2.09 (s, 6H, CH_3_ Ac), 1.86 (s, 3H, CH_3_ Ac), 1.01 (s, 9H, CH_3_ TBDPS). **^13^C NMR (101 MHz, CDCl_3_)** δ 170.3, 169.7 (CO Ac), 164.4 (CO Bz), 153.1 (C2), 151.6, 149.7, 143.0, 143.0, 142.9, 142.9, 141.5, 141.4 (Cq. arom.), 141.4 (C8), 135.7, 135.7 (arom.), 133.7 (Cq. arom.), 133.0 (arom.), 132.9, 132.7 (Cq. arom.), 130.0, 130.0, 129.1, 128.1, 128.0, 128.0, 127.9, 127.3, 127.2, 125.1, 125.1, 125.1 (arom.), 123.3 (Cq. arom.), 120.2, 120.1, 120.1 (arom.), 101.5 (C1’’), 87.0 (C1’), 83.2 (C4’’), 81.2 (d, ^3^*J*_cp_ = 8.1 Hz, C4’), 78.2 (C2’), 71.6 (C2’’), 71.3 (C3’), 70.3 (C3’’), 69.7, 69.6, 69.6 (CH_2_ Fm), 66.2 (d, ^2^*J*_cp_ = 2.0 Hz, C5’), 63.5 (C5’’), 48.0, 48.0, 47.9, 47.9 (CH Fm), 26.9 (CH_3_ TBDPS), 20.9, 20.9, 20.4 (CH_3_ Ac), 19.3 (Cq. TBDPS). **^31^P NMR (162 MHz, CDCl_3_)** δ -1.06 (phosphate). **HRMS** (ESI) [M+H]^+^ calculated for C_72_H_71_N_5_O_15_PSi 1304.44481; found 1304.44407.

*N^6^*-Benzoyl-9-(3’,2”,3”-tri-*O*-acetyl-5’-*O*-(di-fluorenylphosphoryl)-β-parobiosyl)adenine (14)

Phosphotriester **13** (0.33 g, 0.25 mmol) was co-evaporated with 1,4-dioxane (6x) and dissolved in dry THF (2.5 mL). HF·pyridine (~70% HF, 2.25 mL, 25.00 mmol, 100.0 eq) was added at 0 °C and the reaction mixture was stirred at room temperature for 2 hours. Then, the reaction was diluted with DCM and carefully quenched with sat. aq. NaHCO_3_. Next, the aqueous phase was extracted with DCM (1x) and the combined organic layers were washed with H_2_O (1x) and brine (1x), dried over MgSO_4_, filtered and concentrated under reduced pressure. Purification by silica gel column chromatography (0% → 2% MeOH in DCM) afforded compound **14** as a clear oil (0.23 g, 0.22 mmol, 88%). **^1^H NMR (300 MHz, CDCl_3_)** δ 9.46 (s, 1H, NH), 8.81 (s, 1H, H2), 8.27 (s, 1H, H8), 8.03 – 7.98 (m, 2H, arom.), 7.73 – 7.08 (m, 19H, arom.), 6.24 (d, *J* = 6.8 Hz, 1H, H1’), 5.47 (dd, *J* = 5.2, 2.9 Hz, 1H, H3’), 5.28 (d, *J* = 4.6 Hz, 1H, H1’’), 5.14 (dd, *J* = 7.3, 3.6 Hz, 1H, H3’’), 5.02 (t, *J* = 6.4 Hz, 1H, H2’), 4.68 (dd, *J* = 7.3, 4.6 Hz, 1H, H2’’), 4.35 – 4.23 (m, 5H, H4’, CH_2_ Fm), 4.13 – 3.99 (m, 5H, H5’, H4’’, CH Fm), 3.58 – 3.48 (m, 2H, H5’’), 3.41 – 3.27 (m, 1H, 5’’-OH), 2.16 (s, 3H, CH_3_ Ac), 2.09 (s, 3H, CH_3_ Ac), 1.68 (s, 3H, CH_3_ Ac). **^13^C NMR (75 MHz, CDCl_3_)** δ 170.4, 169.7, 169.6 (CO Ac), 164.8 (CO Bz), 153.3 (C2), 151.9, 149.9, 143.0, 142.9, 141.4, 141.4 (Cq. arom.), 141.0 (C8), 133.9 (Cq. arom.), 132.9, 128.9, 128.1, 128.0, 127.3, 127.3, 127.2, 125.1, 125.1, 125.0 (arom.), 122.8 (Cq. arom.), 120.2, 120.1, 120.1 (arom.), 102.1 (C1’’), 85.5 (C1’), 83.0 (C4’’), 81.6 (d, ^3^*J*_cp_ = 7.5 Hz, C4’), 78.3 (C2’), 71.8 (C3’), 71.3 (C2’’), 69.9 (C3’’), 69.8, 69.7 (CH_2_ Fm), 66.5 (d, ^2^*J*_cp_ = 6.0 Hz, C5’), 61.8 (C5’’), 48.0, 48.0, 47.9, 47.9 (CH Fm), 20.9, 20.9, 20.1 (CH_3_ Ac). **^31^P NMR (121 MHz, CDCl_3_)** δ -1.23 (phosphate). **HRMS** (ESI) [M+H]^+^ calculated for C_56_H_53_N_5_O_15_P 1066.32703; found 1066.32694.

*N^6^*-Benzoyl-9-(3’,2”,3”-tri-*O*-acetyl-5’-*O*-(di-fluorenylphosphoryl)-5’’-*O*-(*N,N*-diisopropylamino-*O*-cyanoethyl)phosphoramidite)-β-parobiosyl)adenine (15)

Compound **14** (0.74 g, 0.69 mmol) was co-evaporated with 1,4-dioxane (6x) and dissolved in dry DMF (6.9 mL). DiPEA (0.3 mL, 1.73 mmol, 2.5 eq) and 2-cyanoethyl *N,N*-diisopropylchlorophosphoramidite (170 µL, 0.76 mmol, 1.1 eq) were added and the reaction mixture was stirred at room temperature for 10 minutes. Then, the reaction mixture was quenched with MeOH (100 µL), diluted with EtOAc and the organic phase was washed with sat. aq. NaHCO_3_ (1x), H_2_O (1x) and brine (2x). The organic layer was dried over Na_2_SO_4_, filtered, diluted with toluene and concentrated under reduced pressure. Purification by automated silica gel column chromatography using high-quality IRR silica gel (40-63 µm) (0% → 40% acetone in DCM) afforded phosphoramidite **15** as a white foam and as a mixture of two P(III) diastereomers (0.60 g, 0.47 mmol, 69%). Data for diastereomeric mixture: **^1^H NMR (400 MHz, CDCl_3_)** δ 9.28 (s, 1H, NH), 8.73 (d, *J* = 3.8 Hz, 1H, H2), 8.25 (d, *J* = 5.1 Hz, 1H, H8), 7.97 (d, *J* = 7.9 Hz, 2H, arom.), 7.71 – 7.63 (m, 4H, arom.), 7.55 (t, *J* = 7.4 Hz, 1H, arom.), 7.50 – 7.41 (m, 6H, arom.), 7.37 – 7.15 (m, 8H, arom.), 6.18 (d, *J* = 5.7 Hz, 1H, H1’), 5.50 – 5.43 (m, 1H, H3’), 5.33 – 5.25 (m, 2H, H1’’, H3’’), 5.04 (t, *J* = 5.3 Hz, 1H, H2’), 4.83 (dd, *J* = 7.0, 4.7 Hz, 1H, H2’’), 4.33 – 4.22 (m, 5H, CH_2_ Fm, H4’), 4.18 – 4.15 (m, 1H, H4’’), 4.13 – 4.05 (m, 4H, CH Fm, H5’), 3.82 – 3.65 (m, 4H, O*CH_2_*CH_2_CN, H5’’), 3.57 – 3.49 (m, 2H, (CH_3_)_2_*CH*N), 2.57 (dt, *J* = 16.8, 6.2 Hz, 2H, *CH_2_*CN), 2.16 (s, 3H, CH_3_ Ac), 2.10 (s, 3H, CH_3_ Ac), 1.84 (d, *J* = 8.0 Hz, 3H, CH_3_ Ac), 1.16 – 1.09 (m, 12H, *(CH_3_)_2_*CHN). **^13^C NMR (101 MHz, CDCl_3_)** δ 170.0, 170.0, 169.4, 169.3 (CO Ac), 164.5 (CO Bz), 152.6 (C2), 151.5, 151.4, 149.6, 142.7, 142.6, 142.6 (Cq. arom.), 141.4, 141.3 (C8), 141.2, 141.1, 133.4 (Cq. arom.), 132.6, 128.6, 127.7, 127.7, 127.0, 124.8, 124.8 (arom.), 123.3 (Cq. arom.), 119.9, 119.8 (arom.), 117.7, 117.6 (CN), 101.1, 101.1 (C1’’), 86.6, 86.4 (C1’), 82.1, 82.1, 82.0, 81.9 (C4’’), 81.1, 81.0, 81.0 (C4’), 77.7, 77.6 (C2’), 71.2, 71.1 (C3’), 71.0 (C2’’), 70.0, 69.9 (C3’’), 69.3, 69.3, 69.3, 69.3 (CH_2_ Fm), 66.1, 66.0, 65.9 (C5’), 63.0, 62.9, 62.8 (C5’’), 58.3, 58.2, 58.1, 58.0 (O*CH_2_*CH_2_CN), 47.6, 47.6 (CH Fm), 43.0, 42.9, 42.9, 42.8 ((CH_3_)_2_*CH*N), 24.5, 24.4, 24.4, 24.3 (*(CH_3_)_2_*CHN), 20.6 (CH_3_ Ac), 20.2, 20.1, 20.1, 20.1 (*CH_2_*CN), 20.0 (CH_3_ Ac). **^31^P NMR (162 MHz, CDCl_3_)** δ 149.96, 149.67 (phosphoramidite), -1.12, -1.13 (phosphate). **HRMS** (ESI) [(H-phosphonate)+H]^+^ calculated for C_59_H_57_N_6_O_17_P_2_ 1183.32499; found 1183.32488.

***N*^6^-Benzoyl-3’-*O*-acetyl-5’-*O*-dimethoxytrityl-β-D-adenosine (51A) / *N*^6^-Benzoyl-2’-*O*-acetyl-5’-*O*-dimethoxytrityl-β-d-adenosine (51B) / *N*^6^-Benzoyl-2’, 3’-di-*O*-acetyl-5’-*O*-dimethoxytrityl-β-d-adenosine (52)**

Diol **50** (1.38 g, 2.05 mmol) was dissolved in pyridine (10 mL). Ac_2_O (174 µL, 1.84 mmol, 0.9 eq) was added dropwise at 0 °C and the reaction mixture was stirred at room temperature overnight. Then, the reaction mixture was concentrated under reduced pressure, diluted with EtOAc and the organic phase was washed with H_2_O (1x). The aqueous phase was extracted with EtOAc (3x) and the combined organic layers were dried over MgSO_4_, filtered and concentrated under reduced pressure. Purification by silica gel column chromatography (0% → 5% MeOH in DCM) afforded esters **51A** and **51B** as a white foam and as a regioisomeric mixture (0.62 g, 0.86 mmol, **51A**:**51B** ratio; 0.59:0.41, with an overall yield of 42%) and ester **52** as a white foam (0.35 g, 0.46 mmol, 22%). Data for regioisomeric mixture of **51A** and **51B**: **^1^H NMR (400 MHz, CDCl_3_)** δ 9.25 – 9.11 (m, 1H, NH-AB), 8.79 – 8.65 (m, 1H, H2-AB), 8.25 (s, 0.59H, H8-A), 8.20 (s, 0.41H, H8-B), 8.02 (d, *J* = 7.8 Hz, 2H, arom.-AB), 7.63 – 7.58 (m, 1H, arom.-AB), 7.54 – 7.49 (m, 2H, arom.-AB), 7.43 – 7.38 (m, 1H, arom.-AB), 7.33 – 7.17 (m, 8H, arom.-AB), 6.81 – 6.75 (m, 4H, DMT arom.-AB), 6.28 (d, *J* = 4.7 Hz, 0.41H, H1’-B), 6.09 (d, *J* = 6.7 Hz, 0.59H, H1’-A), 5.88 (t, *J* = 5.1 Hz, 0.41H, H2’-B), 5.47 (dd, *J* = 5.5, 2.1 Hz, 0.59H, H3’-A), 5.13 (t, *J* = 5.9 Hz, 0.59H, H2’-A), 4.86 (t, *J* = 5.1 Hz, 0.41H, H3’-B), 4.40 (q, *J* = 3.2 Hz, 0.59H, H4’-A), 4.27 (q, *J* = 3.8 Hz, 0.41H, H4’-B), 3.78 – 3.75 (m, 6H, CH_3_ DMT-AB), 3.55 – 3.35 (m, 2H, H5’-AB), 2.18 (s, 1.77H, CH_3_ Ac-A), 2.13 (s, 1.23H, CH_3_ Ac-B), 1.95 (bs, 1H, OH-AB). **^13^C NMR (101 MHz, CDCl_3_)** δ 170.5 (CO Ac-A), 170.2 (CO Ac-B), 164.7 (CO Bz-AB), 158.7 (Cq. arom.-AB), 153.0, 152.6 (C2-AB), 151.7 (C4-B), 151.5 (C4-A), 149.8, 149.7 (C6-AB), 144.4, 144.3 (Cq. arom.-AB), 141.7 (C8-B), 141.5 (C8-A), 135.6, 135.6, 135.5, 135.4, 133.6 (Cq. arom.-AB), 133.0, 132.9, 130.2, 130.1, 130.1, 129.0, 129.0, 128.3, 128.1, 128.0, 127.1 (arom.-AB), 123.4 (C5-B), 123.2 (C5-A), 113.3 (arom.-AB), 89.6 (C1’-A), 87.0, 86.9 (Cq. DMT-AB), 86.4 (C1’-B), 84.0 (C4’-B), 83.9 (C4’-A), 76.0 (C2’-B), 74.5 (C2’-A), 74.1 (C3’-A), 70.5 (C3’-B), 63.4, 63.1 (C5’-AB), 55.4 (CH_3_ DMT-AB), 21.0 (CH_3_ Ac-A), 20.8 (CH_3_ Ac-B). **HRMS** (ESI) [M+H]^+^ calculated for C_40_H_38_N_5_O_8_ 716.27149; found 716.27105.

Data for **52**: **^1^H NMR (400 MHz, CDCl_3_)** δ 9.21 (s, 1H, NH), 8.75 (s, 1H, H2), 8.19 (s, 1H, H8), 8.02 (d, *J* = 7.2 Hz, 2H, arom.), 7.61 – 7.57 (m, 1H, arom.), 7.53 – 7.48 (m, 2H, arom.), 7.45 – 7.42 (m, 2H, arom.), 7.34 – 7.20 (m, 7H, arom.), 6.83 – 6.80 (m, 4H, DMT arom.), 6.39 (d, *J* = 6.8 Hz, 1H, H1’), 6.16 (dd, *J* = 6.9, 5.3 Hz, 1H, H2’), 5.72 (dd, *J* = 5.3, 2.8 Hz, 1H, H3’), 4.38 (q, *J* = 3.0 Hz, 1H, H4’), 3.78 – 3.77 (m, 6H, CH_3_ DMT), 3.53 (AB, *J* = 10.7, 3.1 Hz, 1H, H5’), 3.47 (AB, *J* = 10.7, 3.4 Hz, 1H, H5’), 2.15 (s, 3H, CH_3_ Ac), 2.06 (s, 3H, CH_3_ Ac). **^13^C NMR (101 MHz, CDCl_3_)** δ 169.8, 169.5 (CO Ac), 164.8 (CO Bz), 158.7 (Cq. arom.), 153.0 (C2), 152.2 (C4), 149.8 (C6), 144.1 (Cq. arom.), 141.2 (C8), 135.3, 135.3, 133.6 (Cq. arom.), 132.9, 130.2, 128.9, 128.3, 128.1, 128.0, 127.2 (arom.), 123.3 (C5), 113.4 (arom.), 87.2 (Cq. DMT), 84.9 (C1’), 82.8 (C4’), 73.3 (C2’), 71.9 (C3’), 63.1 (C5’), 55.3 (CH_3_ DMT), 20.8, 20.5 (CH_3_ Ac). **HRMS** (ESI) [M+H]^+^ calculated for C_42_H_40_N_5_O_9_ 758.28205; found 758.28200.

***N*^6^-Benzoyl-2’-*O*-hydroquinone-*O,O’*-diacetylhemiester-3’-*O*-acetyl-5’-*O*-dimethoxytrityl-β-d-adenosine (53A) / *N*^6^-Benzoyl-2’-*O*-acetyl-3’-*O*-hydroquinone-*O,O’*-diacetylhemiester-5’-*O*-dimethoxytrityl-β-d-adenosine** **(53B)**

A regiosiomeric mixture of **51A** and **51B** (2.85 g, 3.98 mmol, **51A**:**51B** ratio; 0.5:0.5) was dissolved in pyridine (21 mL). DMAP (49 mg, 0.40 mmol, 0.1 eq), EDC·HCl (0.92 g, 4.78 mmol, 1.2 eq), Et_3_N (0.4 mL, 2.91 mmol, 0.73 eq) and Q-linker (1.08 g, 4.78 mmol, 1.2 eq) were added and the reaction mixture was stirred at room temperature overnight. Then, the reaction mixture was concentrated under reduced pressure, diluted with CHCl_3_ and the organic phase was washed with H_2_O (1x). The aqueous phase was extracted with CHCl_3_ (1x) and the combined organic layers were dried over MgSO_4_, filtered and concentrated under reduced pressure. Purification by automated silica gel column chromatography using high-quality IRR silica gel (40-63 µm) (0% → 10% MeOH in DCM) afforded compounds **53A** and **53B** as a white foam and as a regioisomeric mixture (1.68 g, 1.82 mmol, **53A**:**53B** ratio; 0.5:0.5, with an overall yield of 46%). Data for regioisomeric mixture: **^1^H NMR (400 MHz, CDCl_3_)** δ 8.68 (s, 0.5H, H2-B), 8.67 – 8.58 (m, 1.5H, NH-AB, H2-A), 8.23 (s, 0.5H, H8-B), 8.18 (s, 0.5H, H8-A), 8.06 – 8.02 (m, 2H, arom.-AB), 7.83 (tt, *J* = 7.8, 1.7 Hz, 0.5H, arom.-A/B), 7.58 – 7.53 (m, 1H, arom.-AB), 7.48 – 7.40 (m, 5H, arom.-AB), 7.32 – 7.20 (m, 5.5H, arom.-AB), 6.87 – 6.79 (m, 6H, arom.-AB), 6.74 – 6.67 (m, 2H, arom.-AB), 6.33 (d, *J* = 7.0 Hz, 0.5H, H1’-A), 6.27 (d, *J* = 6.9 Hz, 0.5H, H1’-B), 6.26 – 6.19 (m, 1H, H2’-AB), 5.82 (dd, *J* = 5.0, 2.6 Hz, 0.5H, H3’-B), 5.71 (dd, *J* = 5.2, 2.5 Hz, 0.5H, H3’-A), 4.66 (d, *J* = 1.5 Hz, 1H, CH_2_ Q-AB), 4.59 – 4.52 (m, 3H, CH_2_ Q-AB), 4.39 – 4.36 (m, 1H, H4’-AB), 3.77 – 3.74 (m, 6H, CH_3_ DMT-AB), 3.54 (AB, *J* = 10.8, 2.6 Hz, 1H, H5’-AB), 3.47 (AB, *J* = 10.8, 2.6 Hz, 1H, H5’-AB), 2.11 (s, 1.5H, CH_3_ Ac-A), 1.96 (s, 1.5H, CH_3_ Ac-B). **^13^C NMR (101 MHz, CDCl_3_)** δ 172.5, 172.4 (CO Q carboxylic acid-AB), 169.9 (CO Ac-A), 169.5 (CO Ac-B), 168.3 (CO Q ester-B), 168.1 (CO Q ester-A), 165.5, 165.3 (CO Bz-AB), 158.7 (Cq. arom.-AB), 153.1 (C2-B), 152.9 (C2-A), 152.5 (Cq. arom.-AB), 152.3 (C4-B), 152.1 (C4-A), 149.9 (C6-AB), 147.3 (arom.-AB), 144.1, 144.0 (Cq. arom-AB), 141.7 (C8-B), 141.5 (C8-A), 138.5 (arom.-AB), 135.3, 135.2, 135.2, 135.2, 133.2 (Cq. arom.-AB), 132.9, 130.2, 130.0, 129.2, 128.8, 128.8, 128.3, 128.3, 128.2, 128.1, 127.9, 127.9, 127.2, 124.7 (arom.-AB), 123.5 (C5-B), 123.4 (C5-A), 116.0, 115.9, 115.7, 115.6, 113.4, 113.2, 113.0 (arom.-AB), 87.3, 87.2 (Cq. DMT-AB), 85.1 (C1’-B), 84.9 (C1’-A), 83.0, 82.6 (C4’-AB), 73.8, 72.9 (C2’-AB), 72.7 (C3’-B), 71.8 (C3’-A), 66.0, 65.9, 65.8 (CH_2_ Q-AB), 62.9 (C5’-AB), 55.3 (CH_3_ DMT-AB), 20.7 (CH_3_ Ac-A), 20.4 (CH_3_ Ac-B). **HRMS** (ESI) [M+H]^+^ calculated for C_50_H_46_N_5_O_13_ 924.30866; found 924.30792.

***N*^6^-Benzoyl-2’-*O*-acetyl-3’-Q-CPG-5’-*O*-dimethoxytrityl-β-d-adenosine / *N*^6^-Benzoyl-2’-Q-CPG-3’-*O*-acetyl-5’-*O*-dimethoxytrityl-β-d-adenosine (54)**

 Adenosine derivative **53A** and **53B** (0.38 g, 0.41 mmol, 2.0 eq) was dissolved in DMF (2 mL). PyAOP (0.21 g, 0.41 mmol, 2.0 eq) and DiPEA (142 µL, 0.82 mmol, 4.0 eq) were added and the reaction mixture was stirred at room temperature for 10 minutes. Then, the reaction mixture was added to a 20 mL reaction syringe with filter frit containing aminopropyl-CPG resin (loading 68 µmol/g, 3.00 g, 0.20 mmol, 1.0 eq) in DMF (5.5 mL) and the resulting mixture was shaken at room temperature overnight. The resin was drained and rinsed with DMF (5x) followed by ACN (5x). Then, capping mix (1:2:7 v/v/v, Ac_2_O:pyridine:ACN, 10 mL) was added and the mixture was shaken for 1 hour to cap the remaining unmodified amine groups. The resin was drained, rinsed with ACN (1x) and the cap treatment was repeated (1x). Then, the resin was rinsed with ACN (2x) followed by DMF (2x) and DCM (5x). The CPG resin was dried under N_2_ pressure and the loading was determined by calorimetric trityl analysis at 503 nm. The loading for immobilized adenosine **54** was 36.7 μmol/g (54%).

***N*^6^-Benzoyl-2’-*O*-acetyl-3’-Q-CPG-5’-*O*-(di-fluorenyl)-phosphoryl-β-d-adenosine / *N*^6^-Benzoyl-2’-Q-CPG-3’-*O*-acetyl-5’-*O*-(di-fluorenyl)-phosphoryl-β-d-adenosine (16)**

Functionalized CPG resin **54** (loading 36.7 µmol/g, 1.09 g, ~40 µmol, 1.0 eq) was added to a 20 mL reaction syringe with filter frit. TCA (3% m/v in DCM) was added repeatedly until no yellow color was observed. Then, the resin was rinsed with DCM (3x) followed by ACN (5x) and dried under N_2_ pressure. ETT (0.25 M in ACN, 2.4 mL, 0.60 mmol, 15.0 eq) and bis(9H-fluoren-9-methyl)-diisopropylaminophosphite **21** ^[43]^(0.1 M in ACN, 1.2 mL, 0.12 mmol, 3.0 eq) were added and the resulting mixture was shaken at room temperature for 10 minutes. Then, the resin was drained and the coupling was repeated (2x). Next, the resin was rinsed with ACN (3x). CSO (0.5 M in ACN, 4.0 mL, 2.00 mmol, 50.0 eq.) was added and the mixture was shaken for 5 minutes. The resin was drained and the oxidation was repeated (1x). Then, the resin was rinsed with ACN (5x) and dried under N_2_ pressure to afford immobilized phosphotriester **16**. For analytical purposes, a small portion of immobilized phosphotriester **16** (100 mg, ~3.7 µmol) was added to a 5 mL reaction syringe with filter frit. DBU (10% v/v in ACN, 1 mL, 0.67 mmol, 181 eq.) was added and the mixture was left to stand for 5 minutes. The resin was drained and the DBU treatment was repeated (3x). Then, the resin was rinsed with ACN (5x). NH_4_OH (35%, 3 mL) was added and the reaction mixture was shaken at room temperature overnight to cleave the product from the resin and remove all other protecting groups. Then, the filtrate was collected, concentrated under reduced pressure and used for ^31^P-NMR spectroscopy. **^31^P NMR (162 MHz, D_2_O)** δ 4.53 (phosphate).

**Phosphoadenosyl-ADPr (18)**

Pre-loaded CPG resin **16** (loading 35.4 µmol/g, 282 mg, ~10 µmol, 1.0 eq) was placed in a Mermade 6 oligonucleotide synthesizer and the complete synthesis was performed under an argon atmosphere. Cycle A was performed (1x).

**Cycle A**

The resin was rinsed with ACN (5x). DBU (10% v/v in ACN, 2 mL, 1.34 mmol, 133.7 eq.) was added and the mixture was left to stand for 5 minutes. The resin was drained and the DBU treatment was repeated (3x). Then, the resin was rinsed with ACN (3x) and phosphoramidite **15** (0.1 M in ACN, 300 µL, 30 µmol, 3.0 eq) and ETT (0.25 M in ACN, 600 µL, 150 µmol, 15.0 eq) were added. The mixture was left to stand for 10 minutes, drained and the coupling was repeated (2x). Next, the resin was rinsed with ACN (3x). CSO (0.5 M in ACN, 1 mL, 500 µmol, 50.0 eq.) was added and the mixture was left to stand for 5 minutes. The resin was drained and the oxidation was repeated (1x).

After Cycle A, the resin was rinsed with ACN (3x). DBU (10% v/v in ACN, 2 mL, 1.34 mmol, 133.7 eq.) was added and the mixture was left to stand for 5 minutes. The resin was drained and the DBU treatment was repeated (3x). Then, the resin was rinsed with ACN (3x). NH_4_OH (35%, 10 mL) was added and the reaction mixture was shaken at room temperature overnight to cleave the product from the resin and remove all the protecting groups. Then, the filtrate was collected, concentrated under reduced pressure and purification by HW-40 gel filtration (0.15 M NH_4_OAc in 10% ACN/MilliQ) afforded phosphoadenosyl-ADPr **18** as a white solid (1.30 mg, 1.36 µmol, 13.6%) after repeated lyophilization.

**^1^H NMR (500 MHz, D_2_O)** δ 8.42 (s, 1H, H2), 8.41 (s, 1H, H2), 8.19 (s, 1H, H8), 8.18 (s, 1H, H8), 6.19 (d, *J* = 4.0 Hz, 1H, H1’-A), 6.01 (d, *J* = 6.0 Hz, 1H, H1’-C), 5.25 (d, *J* = 4.1 Hz, 1H, H1-B), 4.68 (dd, *J* = 6.0, 5.1 Hz, 1H, H2’-C), 4.65 (dd, *J* = 5.3, 4.0 Hz, 1H, H2’-A), 4.55 (t, *J* = 5.3 Hz, 1H, H3’-A), 4.48 (dd, *J* = 5.1, 3.3 Hz, 1H, H3’-C), 4.39 – 4.36 (m, 1H, H4’-A), 4.35 – 4.33 (m, 2H, H4-B, H4’-C), 4.24 – 4.18 (m, 5H, H5’-A, H2-B, H3-B, H5-B), 4.13 – 4.09 (m, 1H, H5’-A), 4.07 – 4.03 (m, 2H, H5’-C). **^13^C NMR (126 MHz, D_2_O)** δ 154.2, 154.1 (C4-A, C4-C), 151.2, 151.0 (C8-A, C8-C), 148.6, 148.3 (C6-A, C6-C), 140.2, 140.1 (C2-A, C2-C), 118.4, 118.3 (C5-A, C5-C), 101.5 (C1-B), 87.1 (C1’-C), 86.6 (C1’-A), 84.2, 84.2, 84.0, 84.0 (C4-B, C4’-C), 83.5 (d, ^3^*J*_cp_ = 8.8 Hz, C4’-A), 79.1 (C2’-A), 74.5 (C2’-C), 71.3 (C2-B), 70.5 (C3’-C), 69.8 (C3-B), 69.5 (C3’-A), 65.6 (d, ^2^*J*_cp_ = 5.0 Hz, C5’-C), 65.2 (d, ^2^*J*_cp_ = 2.5 Hz, C5-B), 63.8 (d, ^2^*J*_cp_ = 3.8 Hz, C5’-A). **^31^P NMR (202 MHz, D_2_O)** δ 0.33 (phosphate), -11.20, -11.31, -11.37, -11.47 (pyrophosphate). **LC-MS** (0→50% ACN [0.1% TFA]): Rt = 2.5 min, m/z: 889.2. **HRMS** (ESI) [M+H]^+^ calculated for C_25_H_36_N_10_O_20_P_3_ 889.13147; found 889.13112.

**3. Characterization Data**

**Figure S4** ^1^H NMR Spectrum of Compound **6**

 **Figure S5** ^1^H NMR Spectrum (presat) of Compound **6**

**Figure S6** ^31^P NMR Spectrum of Compound **6**

**Figure S7** ^1^H NMR Spectrum of Compound **7**

**Figure S8** ^1^H NMR Spectrum (presat) of Compound **7**

**Figure S9** ^31^P NMR Spectrum of Compound **7**

**Figure S10** ^1^H NMR Spectrum of Compound **8**

**Figure S11** ^1^H NMR Spectrum (presat) of Compound **8**

**Figure S12** ^31^P NMR Spectrum of Compound **8**


**Figure S13** ^1^H NMR Spectrum of Compound **9**

 **Figure S14** ^1^H NMR Spectrum (presat) of Compound **9**

**Figure S15** ^31^P NMR Spectrum of Compound **9**

**Figure S16** ^1^H NMR Spectrum of Compound **10** **Figure S17** ^1^H NMR Spectrum (presat) of Compound **10**

**Figure S18** ^31^P NMR Spectrum of Compound **10**

**Figure S19** ^1^H NMR Spectrum of Compound **40**

**Figure S20** ^13^C NMR Spectrum of Compound **40**

**Figure S21** ^1^H NMR Spectrum of Compound **40**

**Figure S22** ^13^C NMR Spectrum of Compound **40**

**Figure S23** ^1^H NMR Spectrum of Compound **41**

**Figure S24** ^13^C NMR Spectrum of Compound **41**

**Figure S25** ^1^H NMR Spectrum of Compound **42**

**Figure S26** ^13^C NMR Spectrum of Compound **42**

**Figure S27** ^1^H NMR Spectrum of Compound **44**

**Figure S28** ^13^C NMR Spectrum of Compound **44**

**Figure S29** ^1^H NMR Spectrum of Compound **45**

**Figure S30** ^13^C NMR Spectrum of Compound **45**

**Figure S31** ^1^H NMR Spectrum of Compound **46**

**Figure S32** ^13^C NMR Spectrum of Compound **46**

**Figure S33** ^1^H NMR Spectrum of Compound **48**

**Figure S34** ^13^C NMR Spectrum of Compound **48**

**Figure S35** ^1^H NMR Spectrum of Compound **49**

**Figure S36** ^13^C NMR Spectrum of Compound **49**

**Figure S37** ^1^H NMR Spectrum of Compound **11**

**Figure S38** ^13^C NMR Spectrum of Compound **11**

**Figure S39** ^1^H NMR Spectrum of Compound **12**

**Figure S40** ^13^C NMR Spectrum of Compound **12**

**Figure S41** ^1^H NMR Spectrum of Compound **13**

**Figure S42** ^13^C NMR Spectrum of Compound **13**

**Figure S43** ^31^P NMR Spectrum of Compound **13**

**Figure S44** ^1^H NMR Spectrum of Compound **14**

**Figure S45** ^13^C NMR Spectrum of Compound **14**

**Figure S46** ^31^P NMR Spectrum of Compound **14**

**Figure S47** ^1^H NMR Spectrum of Compound **15**

**Figure S48** ^13^C NMR Spectrum of Compound **15**

**Figure S49** ^31^P NMR Spectrum of Compound **15**

**Figure S50** ^1^H NMR Spectrum of Compound **51**

**Figure S51** ^13^C NMR Spectrum of Compound **51**

**Figure S52** ^1^H NMR Spectrum of Compound **52**

**Figure S53** ^13^C NMR Spectrum of Compound **52**

**Figure S54** ^1^H NMR Spectrum of Compound **53**

**Figure S55** ^13^C NMR Spectrum of Compound **53**

**Figure S56** ^31^P NMR Spectrum of Compound **16** (crude, upon cleavage from the resin)

**Figure S57** ^1^H NMR Spectrum of Compound **18**

**Figure S58** ^1^H NMR Spectrum (presat) of Compound **18**

**Figure S59** ^13^C NMR Spectrum of Compound **18**

**Figure S60** ^31^P NMR Spectrum of Compound **18**
